# Myeloperoxidase and Other Markers of Neutrophil Activation Associate With Malaria and Malaria/HIV Coinfection in the Human Placenta

**DOI:** 10.3389/fimmu.2021.682668

**Published:** 2021-10-19

**Authors:** Demba Sarr, Lilian J. Oliveira, Brittany N. Russ, Simon O. Owino, Joab D. Middii, Stephen Mwalimu, Linda Ambasa, Faris Almutairi, John Vulule, Balázs Rada, Julie M. Moore

**Affiliations:** ^1^ Department of Infectious Diseases, College of Veterinary Medicine, University of Georgia, Athens, GA, United States; ^2^ Department of Pathology, College of Veterinary Medicine, University of Georgia, Athens, Georgia, United States; ^3^ Department of Infectious Diseases and Immunology, College of Veterinary Medicine, University of Florida, Gainesville, FL, United States; ^4^ Vector Biology and Control Research Centre, Kenya Medical Research Institute, Kisian, Kenya; ^5^ University of Georgia/Kenya Medical Research Institute Placental Malaria Study, Siaya District Hospital, Siaya, Kenya; ^6^ Faculty of Science, Department of Zoology, Maseno University, Maseno, Kenya; ^7^ Kisumu Specialists Hospital Laboratory, Kisumu, Kenya; ^8^ Animal and Human Health Program, International Livestock Research Institute, Nairobi, Kenya; ^9^ #1 Heartsaved Adult Family Care, Marysville, WA, United States; ^10^ Department of Pharmaceutical and Biomedical Sciences, College of Pharmacy, University of Georgia, Athens, GA, United States

**Keywords:** neutrophils, pregnancy, NETs (neutrophil extracellular traps), placental malaria, myeloperoxidase, *Plasmodium falciparum*

## Abstract

**Introduction:**

Placental malaria (PM) is characterized by accumulation of inflammatory leukocytes in the placenta, leading to poor pregnancy outcomes. Understanding of the underlying mechanisms remains incomplete. Neutrophils respond to malaria parasites by phagocytosis, generation of oxidants, and externalization of Neutrophil Extracellular Traps (NETs). NETs drive inflammation in malaria but evidence of NETosis in PM has not been reported. Neutrophil activity in the placenta has not been directly investigated in the context of PM and PM/HIV-co-infection.

**Methods:**

Using peripheral and placental plasma samples and placental tissue collected from Kenyan women at risk for malaria and HIV infections, we assessed granulocyte levels across all gravidities and markers of neutrophil activation, including NET formation, in primi- and secundigravid women, by ELISA, western blot, immunohistochemistry and immunofluorescence.

**Results:**

Reduced peripheral blood granulocyte numbers are observed with PM and PM/HIV co-infection in association with increasing parasite density and placental leukocyte hemozoin accumulation. In contrast, placental granulocyte levels are unchanged across infection groups, resulting in enhanced placental: peripheral count ratios with PM. Within individuals, PM- women have reduced granulocyte counts in placental relative to peripheral blood; in contrast, PM stabilizes these relative counts, with HIV coinfection tending to elevate placental counts relative to the periphery. In placental blood, indicators of neutrophil activation, myeloperoxidase (MPO) and proteinase 3 (PRTN3), are significantly elevated with PM and, more profoundly, with PM/HIV co-infection, in association with placental parasite density and hemozoin-bearing leukocyte accumulation. Another neutrophil marker, matrix metalloproteinase (MMP9), together with MPO and PRTN3, is elevated with self-reported fever. None of these factors, including the neutrophil chemoattractant, CXCL8, differs in relation to infant birth weight or gestational age. CXCL8 and MPO levels in the peripheral blood do not differ with infection status nor associate with birth outcomes. Indicators of NETosis in the placental plasma do not vary with infection, and while structures consistent with NETs are observed in placental tissue, the results do not support an association with PM.

**Conclusions:**

Granulocyte levels are differentially regulated in the peripheral and placental blood in the presence and absence of PM. PM, both with and without pre-existing HIV infection, enhances neutrophil activation in the placenta. The impact of local neutrophil activation on placental function and maternal and fetal health remains unclear. Additional investigations exploring how neutrophil activation and NETosis participate in the pathogenesis of malaria in pregnant women are needed.

## Introduction

Malaria infection during pregnancy is a significant public health problem with substantial effects on the mother, her fetus, and the newborn child [reviewed by (1)]. Accumulation of parasites in the placenta is a common feature of *Plasmodium falciparum* infection in pregnant women, mediated by VAR2CSA, a parasite protein exported to the surface of infected red blood cells, which binds to chondroitin sulfate A on proteoglycans, including syndecan-1 (2), in the placenta. Paucigravid women (in the first or second pregnancy) are especially vulnerable and are more likely than multigravidae (three or more pregnancies) and nonpregnant women to develop severe malaria (1). The interplay between parasites and the placenta is associated with inflammation characterized by the recruitment, retention, and activation of innate immune cells including polymorphonuclear leukocytes (neutrophils) (3) and is known as placental malaria (PM) (4). PM is associated with adverse pregnancy outcomes such as maternal anemia, stillbirth, and low birth weight (LBW) due to intrauterine growth restriction, and is most severe in the first pregnancy (5). How neutrophils in particular may affect placental pathology and fetal growth in the context of malaria remains a mystery.

Previous studies addressing potential interactions between PM and HIV infection suggest that the latter impairs immunity against malaria (6). HIV-infected pregnant women have more frequent and higher density parasitemia than HIV-negative pregnant women (7–11). Importantly, malaria accelerates HIV disease progression and higher viral load among pregnant women. Fetal complications in PM and association between maternal HIV status and fetal outcome have also been addressed (11–17). A definitive role for neutrophils in pathogenesis of PM alone and co-infections with HIV has been rarely studied. In one study, the number of circulating pigmented (Hz-bearing) neutrophils negatively correlated with birth weight, suggesting that these cells may have a pathogenic role in PM and thus may serve as prognostic markers for malaria-associated low birth weight (18). Another study reported that circulating neutrophils were reduced in pregnant women with *P. falciparum* malaria compared to negative controls (3). Others have found elevated neutrophil levels in placental relative to peripheral blood in malaria-infected women (19). Limited studies that performed direct measures have noted increased neutrophil levels by placental histopathology (20, 21). Consistent with this, cytokines and chemokines that can attract neutrophils, namely MIF, CXCL8/IL-8, and CCL3, are elevated in human PM (22–27).

Neutrophils are essential effector cells of the innate immune system. In humans, neutrophils are the most abundant type of white blood cell, accounting for 70% of all leukocytes in the blood of healthy adults (28). During pregnancy, the neutrophil count begins to increase in the second month and plateaus in the second or third trimester, a time at which the total number of white blood cells ranges from 9,000 to 15,000 X10^6^/L (29). These cells are classically considered to be short-lived and act as the first line of defense in innate immunity, ensuring tissue restitution following resolution of infection (30–32). Neutrophils can rapidly be recruited to sites of infection and tissue injury (33), where they generate reactive oxygen species (ROS) through the activity of NADPH oxidase, thereby initiating antibacterial/antiparasitic defense (34). Neutrophils clear infections by phagocytosis, generation of ROS, release of potent bactericidal enzymes by degranulation, and formation of neutrophil extracellular traps (NETs) (35).

As evidenced in malaria, however, neutrophils represent a double-edged sword. These cells are activated and are capable of clearing malaria parasites by a variety of mechanisms (reviewed by (36), yet they are implicated in pathogenic mechanisms as well (37). Mice developing malaria-associated acute lung injury/acute respiratory distress syndrome (ALI/ARDS) had greater neutrophil accumulation in the lungs compared to mice that did not develop pulmonary complications (38). In these mice, targeting of neutrophils decreased the development of malaria-associated ALI/ARDS and significantly increased mouse survival (38), suggesting that neutrophils play a significant role in the pathogenesis of ALI/ARDS during experimental severe malaria and could be targeted to improve disease outcome.

Oxidative damage to tissues is also a key attribute of malaria pathogenesis that may be in part attributable to neutrophils. Retinopathy-positive cerebral malaria is associated with accumulation of neutrophils (39). Likewise, previous work with *P. chabaudi* and *P. berghei* ANKA infection in mice indicated that neutrophils were responsible for liver damage, cerebral complications, and ALI/ARDS (38, 40, 41). Uptake of hemozoin (Hz)-containing digestive vacuoles by neutrophils drives a rapid oxidative burst but suppresses subsequent neutrophil activity (42). Oxidative damage in PM has been reported in humans and in mouse models, but key drivers remain unclear (43–46).

NETs are generated by the extrusion of DNA strands into the extracellular milieu, where they can entrap invasive pathogens (47, 48). The most common method for NET detection *in vitro* is microscopic observation (49), with immunodetection of neutrophil-derived proteins such as myeloperoxidase (MPO) and proteinase 3 (PRTN3) (50, 51) colocalized with DNA (49). NETs in tissue samples have similarly been shown as extracellular DNA colocalized with neutrophil-derived proteins (49). NET remnants such as DNA and neutrophil-derived protein complexes (MPO-DNA; neutrophil elastase (NE)-DNA) and citrullinated histones can be determined by ELISA in fluid samples (50, 51) or detected by flow cytometry (52, 53) as indicators of NETosis.

A role for NETosis in both protection and pathogenesis in malaria is emerging. *P. falciparum*-infected red blood cells reportedly stimulate human neutrophils to release NETs *in vitro* (38). The latest mechanistic investigations of NETs in malaria show that they are released by neutrophils exposed to malaria parasites and impede parasite spread thereby controlling infection (54). Furthermore, these studies provide evidence that NET release in malaria is independent of cell-cell contact and is mediated by macrophage migration inhibitory factor and peptidylarginine deiminase 4 (PAD4)-dependent histone citrullination (54). Interestingly, malaria parasite species have been shown to produce DNase that degrades NETs and the deficiency of this enzyme resulted in lower parasitemia in mice (55). Importantly, NETosis has been linked to severe malaria in human infection and in mouse models (38, 56–58). Using human samples and a mouse model for malaria, Knackstedt et al. demonstrated that heme-induced NETs are essential for malaria pathogenesis, with granulopoiesis and endothelial cell activation as two mechanisms of NET-mediated inflammation of the vasculature (58).

The present study investigates granulocyte levels and neutrophil activity in the peripheral and placental blood and tissue of parturient Kenyan women exposed to malaria and HIV. Neutrophils and associated markers appear to be influenced by these infections and preliminary evidence of NETosis in the placenta blood is offered. These data show a potential implication of neutrophils in the pathogenesis of PM but further studies are required to characterize the mechanisms by which this occurs.

## Material and Methods

### Ethics Statement

The study supporting collection of samples used in this report was approved by the Kenya Medical Research Institute, the Centers for Disease Control and Prevention, and the University of Georgia Institutional Review Boards. All study participants provided written informed consent before enrollment and procedures and instruments involving human subjects, sample collection and data analysis, processing, and testing were approved throughout the conduct of patient recruitment. All samples and data are anonymized.

### Study Participants and Sample Collection

Participant recruitment and sample collection have been previously described (11, 59) (Matthias et al., manuscript in preparation). Briefly, the recruitment of 222 participants was performed at New Nyanza Provincial General Hospital, a public referral hospital, in Kisumu from November, 2002 to May, 2004. Subsequently, 825 participants were recruited at Siaya District Hospital, a public secondary health facility in Siaya until September, 2008. Women of all gravidities and uncomplicated vaginal deliveries were randomly recruited from patients admitted to the Delivery Ward of these hospitals. Only women with no health issues aside from malaria or HIV were eligible for full participation in the study. Maternal demographic and clinical information was collected and summarized, including whether or not participants self-reported fever within the two weeks prior to delivery. Infant gestational age was estimated using the modified Dubowitz score, and birth weight in grams was measured within eight hours after delivery. Maternal placental (intervillous) blood (IVB) was collected by the prick method within five minutes of placental expulsion (60). Peripheral blood was collected by venipuncture of the cubital vein within 12 hours post-partum. Platelet-free plasma was prepared as described (59) and stored continuously at -80°C, avoiding multiple cycles of freeze-thaw. Complete blood count (CBC) of both peripheral blood and IVB to estimate total white blood cell (WBC) and granulocyte counts was performed simultaneously with a Beckman Coulter AcT diff2 (Beckman Coulter Corporation, Miami, FL) within eight hours of blood collection. Although the majority of granulocytes detected by CBC are expected to be neutrophils, differential analysis for granulocytes was not available; thus, granulocyte levels are reported for this study. Full thickness placental tissue sections were collected from three unique regions of the placental disk, and fixed in Streck Tissue Fixative (Streck Inc., Omaha, NE). Five-micron sections were stained with hematoxylin and eosin for histopathological examination or left unstained for immunohistochemical analysis. From the same placental regions, 125 mm^3^ sections of villus tissue underlying the placental basal plate were collected and flash frozen in liquid nitrogen for future molecular analysis and with Tissue-Tek OCT compound (Sakura Finetek USA, Inc., Torrance, CA) for immunofluorescence assessments and stored continuously at ≤-80°C until use.

Parasitemia was assessed on thick and thin smears of peripheral and placental blood and estimation of thick smear parasite density assumed 8,000 WBCs per µL of blood for both peripheral and placental blood. Percentage of leukocytes in the maternal placental vascular space bearing Hz was calculated from these thick smears. HIV serostatus was determined by rapid tests as previously described (11, 59).

### Study Designs

**Figure 1:**
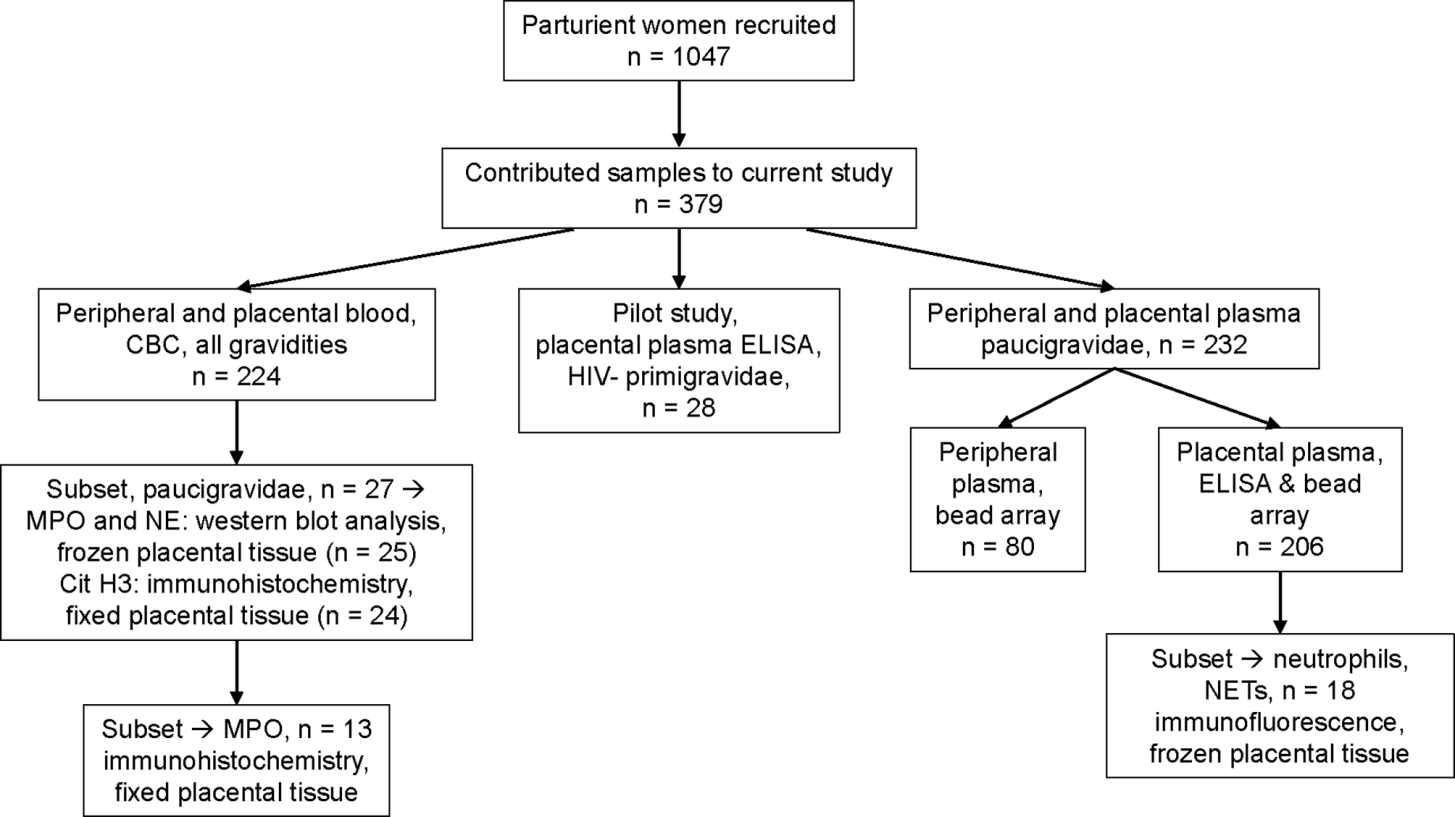
Diagram of sample collection and testing. Shown are the number of patients recruited in a large cohort of parturient women in western Kenya, and samples contributed and tested in this study. Details of sample selection criteria are described in Materials and methods. Overlap of sample assessment on a per patient basis is depicted in **Supplemental Figure 1**.

Among the 1047 women recruited into the study, samples and data from a subset of 379 women are included in the work described here. Sample selection and use are summarized in **Figure 1** and **Supplemental Figure 1**, and patient characteristics are summarized in **Table 1**. All malaria infections are attributable to *P. falciparum;* a single participant was diagnosed with a *P. falciparum/P. malariae* mixed infection. Paired peripheral and placental data from Complete Blood Count were available from 224 women (79 primigravidae, 54 secundigravidae and 91 multigravidae, representing four infection groups (uninfected, malaria only (PM+HIV-), HIV only (PM-HIV+), and co-infected (PM+HIV+)). Gravidity differs significantly across the infection groups providing CBC data (*P=*0.0093 by Kruskal-Wallis test with post-hoc group-wise comparisons by Dunn’s multiple comparisons test; median, interquartile range (IQR): PM-HIV-, 2, 1 – 3; PM+HIV-, 1, 1 - 2; PM-HIV+, 3, 2 – 4; PM+HIV+, 2, 1 – 3). Subsequent analyses focused on primigravid and secundigravid (paucigravid) women who experience the most significant outcomes with malaria and HIV infections in this setting (10, 61) (Matthias et al., manuscript in preparation).

**Table 1:** Descriptive characteristics of study population stratified by gravidity.

Characteristics	All Women (n = 379)	Paucigravid (n = 288)	Multigravid^a^ (n = 91)	*P*-value^b^
**Maternal Sociodemographic**				
Gravidity	1 (1-11)	1 (1-2)	4 (3-11)	-
Primigravid (%)	192 (50.6)	192 (66.7)	-	-
Age (years)	21 (13-39)	19 (13-32)	26 (20-39)	<0.0001
Married (%)	258 (68.1)	171 (59.4)	87 (95.6)	<0.0001
Luo ethnicity (%)	348 (91.8) (n=377)	259 (90.5) (n=286)	89 (97.8)	0.0230
Siaya^c^ residence (%)	342 (90.2)	251 (87.1)	91 (100)	<0.0001
**Laboratory**				
Fever^d^ at admission (%)	4 (3.3) (n=123)	3 (3.1) (n=97)	1 (3.8) (n=26)	1
HIV^e^ seropositive* (%)	103 (27.2)	75 (26)	28 (30.8)	0.4177
Malaria smear positive* (%)	131 (34.6)	113 (39.2)	18 (19.8)	0.0006
Parasite density/µL^f^ (range; interquartile range)	4,976 (40-226,208; 618-25,345) (n=131)	5,208 (40-226,208; 958-31,188) (n=113)	2,433 (83-119,107; 479-7,521) (n=18)	0.0752
Peripheral hemoglobin^g^ (g/dL)	11.3 (5.30-20.4) (n=319)	11.3 (5.30-20.2) (n=228)	11.4 (5.80-20.4) (n=91)	0.5433
Placental hemozoin load^h^	3.5 (0.3-82) (n=127)	3.7 (0.3-82) (n=108)	1.6 (0.6-54) (n=19)	0.1820
**Newborn**				
Birth weight (g)	3,200 (2,000-4,500)	3,000 (2,000-4,400)	3,400 (2,600-4,500)	<0.0001
Low birth weight (≤ 2500 g) (%)	61 (16.1)	61 (21.2)	0 (0)	<0.0001
Gestational age (weeks)	38 (34-42) (n=376)	38 (34-40) (n=285)	38 (35-42)	0.0116
Preterm birth (<37 weeks) (%)	56 (14.9) (n=376)	49 (17.2) (n=285)	7 (7.7)	0.0277
Male infant (%)	193 (50.9)	150 (52.1)	43 (47.2)	0.4709
**Self-reported history**				
Fever, past two weeks^i^ (%)	77 (20.3)	63 (21.9)	14 (15.4)	0.2315

Data are shown as number (percent) or median (range) unless otherwise noted. Sample sizes are shown where missing data reduce group numbers for specific parameters or only a subset of the group have values >0.

a Multigravidae contribute complete blood count data only;

b Comparison of paucigravidae with multigravidae by two-tailed Fisher’s exact test (proportions), Mann Whitney test (continuous data) or Welch’s t test (log transformed continuous data).

c remainder were recruited in Kisumu;

d defined as >37.6°C.

e HIV, human immunodeficiency virus;

f parasitemia measured in placental blood, analysis done on log-transformed data;

g by complete blood count.

h percent white blood cells observed on placental blood thick smear with engulfed hemozoin;

i self-reported fever or malaria in the last two weeks;

*factors used to guide sample selection.

Pilot data to detect markers of neutrophil activation and NETosis (cell-free DNA, DNA-human neutrophil elastase (NE) complexes, histones) in placental plasma were generated using HIV seronegative primigravid placental plasma (**Figure 1**), selected on the basis of placental histopathological status (uninfected, and infected: acute, chronic, chronic inflammatory). Acute infection is defined as the presence of infected red blood cells (iRBC), white blood cell (WBC) count by CBC <13,000/uL, and hemozoin scores [as described in (Avery et al., 2012)] ≤1 in WBCs and in fibrin. Chronic infection is defined as presence of iRBC, WBC count <13,000/uL, and hemozoin scores ≥2 in WBCs and in fibrin. Chronic inflammatory infection is defined as presence of iRBC, WBC count >13,000/uL, and hemozoin scores ≥2 in WBCs and in fibrin. Uninfected samples were confirmed parasite PCR negative and lacked iRBCs in histological sections. Subsequent ELISA data were generated from paucigravid women (**Figure 1**), representing malaria and HIV positive and negative women, with balanced selection of primigravid and secundigravid women within each infection group (PM-HIV-, PM+HIV-, PM-HIV+, PM+HIV+), and with matched selection across groups of infant birth weights, in both cases to the extent that sample availability allowed. Frozen placental tissues for immunofluorescence were selected to represent each infection group from among samples with MPO data, based on sample availability (**Figure 1**). Immunohistochemistry and western blot experiments utilized paucigravid samples (**Figure 1**), representing the four infection groups, with matched selection for granulocyte count (by CBC) ranges across each group.

### ELISA

Levels of myeloperoxidase (MPO), proteinase 3 (PRTN3), matrix metalloproteinase (MMP9), and CXCL8 were quantified in peripheral and placental blood using a commercial ELISA kit (R&D Systems, DuoSet, Minneapolis, MN) or bead arrays (R&D Systems) following manufacturer instructions and as previously described (62, 63). The experimental lower limits of detection for MPO were 125 pg/ml (ELISA) and 130 pg/mL (bead array), 125 pg/mL for MMP9, 10 pg/mL for PRTN3 and 3.9 pg/mL for CXCL8. Citrullinated histone H3 was measured using a kit from Cayman Chemical (Ann Arbor, MI), with a lower limit of detection at 0.1 ng/mL. Levels of NE-DNA complexes were assessed using an in-house protocol established to detect NET formation (64). In this assay, rabbit anti-NE (1:2,000, Calbiochem, San Diego, CA) was used as capture antibody and horseradish peroxidase-conjugated anti-DNA antibody (1:500, Roche, Indianapolis, IN) as detection antibody. Cell-free double stranded DNA was detected using the Quant-iT™ PicoGreen™ dsDNA Assay Kit (ThermoFisher Scientific, Grand Island, NY, USA) according to the manufacturer’s instructions. DNA concentrations were quantitated using a known DNA standard with a lower limit of detection of 5 ng/mL as previously described (65). Total histone H3 levels were detected using a commercial kit (Active Motif, Carlsbad, CA) according to manufacturer instructions; the lower limit of detection was 150 ng/mL.

### Immunohistochemistry and Immunofluorescence

Unstained 5 μm placental tissue sections were dewaxed for 15 minutes at 65°C followed by two incubations in xylene (2x5 minutes). Sections were then rehydrated in alcohol and antigen-retrieved with Sodium Citrate Buffer as previously described (66). Sections were then brought to room temperature and exposed to endogenous peroxidase activity block (DAKO, Cat#S2023) for 15 minutes. After washing with 1X TBST, sections were incubated with 10% Goat Serum for 10 minutes and incubated with the primary antibody (1/500 for MPO or 1/500 for citH3 in 1% Goat Serum) overnight at 4°C. The next day, samples were washed with 1X TBST (3x5 minutes) and incubated with polymer HRP anti-rabbit IgG for 30 minutes at RT. After three washes in 1X TBST, sections were exposed to DAB for 5 minutes, washed with distilled water, counterstained with hematoxylin (Cat#H3401-500, Vector Laboratories), dehydrated, and mounted with acrytol mounting medium (Cat#13518, Electron Microscopy Sciences, Hatfield, USA).

Immunofluorescent analysis was performed on 5 µm cryo-sections of OCT-preserved placental tissue. The sections were fixed in ice-cold acetone for 10 minutes and air dried for 1 hour. Following a rehydration step with 0.05 mM Tris-buffered saline (TBS; pH 7.5), sections were incubated with Protein Block, Serum-Free solution from Agilent DAKO (X0909, Santa Clara, CA, USA) for 1 hour. Rabbit anti-human MPO was purchased from Agilent DAKO (A039829-2, Santa Clara, CA, USA) and rabbit -anti-NE was purchased from Millipore-Sigma (481001, Burlington, MA, USA). Antibodies were pre-labeled using the Zenon Alexa Fluor 488 rabbit IgG1 labeling kit for anti-human MPO and Zenon Alexa Fluor 594 rabbit IgG1 labeling kit for anti-NE from Thermo Fisher Scientific (Waltham, MA USA). The labeled antibodies were diluted in Antibody Diluent reagent from Agilent DAKO (X0909, Santa Clara, CA, USA) at 1:100 and the sections incubated overnight a 4° C. Negative controls were labelled with an irrelevant prelabeled isotype control at the same concentration and same labeling as the primary antibody. The sections were then washed three times in TBS and labeled with Hoescht 33342 reagent (2.3 μg/ml) for 15 minutes. The slides were washed again, and cover slips were mounted using Prolong Antifade mounting medium (Thermo Fisher Scientific, Waltham, MA USA).

Slides were examined using a Leica DM2500 LED microscope with filters 02 (DAPI filter), 03 (FITC filter) and 15 (rhodamine filter) at 20 X magnification. Digital images were acquired using Leica LASX software and a high-resolution Leica DMC6200 digital camera. Five random fields within each villi section were captured for morphometric analysis within a 197.7 mm^2^ field of view (FOV). A total cell number was generated and analyzed for each sample based on the sum of the average number of cells per FOV in each sample.

For image analysis of immunohistochemistry samples, several random FOV were acquired in RBG format and exported as tag image format (*tif) with the respective metadata. Using QuPath v0.2.3‐m4 software (67), the digital images were preprocessed using the built-in visual stain editor to estimate and adjust stain vectors to improve staining quality. The round cells in the intervillous spaces were manually annotated based on hematoxylin filter to warrant the selection of all nucleated cells in the analyzed FOV. The intensity of MPO staining was measured as optical density (OD) and classified by the module “positive cell detection” using adjusted pixel size (0.1465 µM) to match the image resolution and automatic thresholds. The cell detection measurements were compiled. The number of positive cells and the intensity of DAB staining (per mm^2^) were used in the statistical analysis. All analyzed images were blindly evaluated by an observer for quality control purposes prior to data export. Due to the variability of preservation of intervillous blood among the samples, the minimum for evaluation was three FOV or at least 100 intervillous round cells.

Analysis of immunofluorescence samples was also performed using QuPath v0.2.3‐m4 software (67). Images of three fluorescence channels (green, red and blue) were overlaid. The positive cells for either MPO or NE or dually positive cells were manually annotated. The annotated cells were always associated with blue stained nuclei (DNA material). The fluorescence intensity of MPO staining was measured in the green channel and intensity of NE staining was measured in the red channel using the “Analysis” and “Calculate features” with automatic thresholds. Additional overlays of negative controls were exported, and random areas were analyzed to set a threshold of nonspecific fluorescence by average of intensity of fluorescence in these areas in the green and red channels. An annotated cell was considered positive when the fluorescence intensity was higher than the average fluorescence intensity in either or both of the green and red channels in the negative controls. NETs were identified as dual positive for MPO and NE, associated with DNA staining, and with a size higher than 102 µM^2^ as reported (68). All analyzed images were blindly evaluated by an observer for quality control purposes prior to data export.

### Western Blotting

Placental villous tissue (30 mg frozen weight) was homogenized in RIPA buffer supplemented with proteinase inhibitor cocktail. Samples were homogenized with Tissue Lyser (Qiagen, Valencia, USA) and centrifuged at 10,000g for 15 minutes. Protein concentrations were determined by bicinchoninic acid (BCA) method (Thermo Scientific, Rockford, USA) with bovine serum albumin (BSA) as a standard. Equal individual protein samples were prepared and stored at -80°C until use. Proteins (30 μg/sample) were separated by SDS-PAGE, blotted onto nitrocellulose membranes (Biorad, Hercules, USA) and probed with monoclonal or polyclonal rabbit antibodies specific for MPO (Agilent Technology, Santa Clara, USA), NE (Abcam, Cambridge, USA), citrullinated histone H3 (Abcam), and Hsp 90 (Cell Signaling Technologies) (as a loading control). Overnight incubation with primary antibody at 4°C was followed by one-hour incubation with anti-rabbit horseradish peroxidase secondary conjugates (Vector Laboratories). Proteins were detected using an enhanced chemiluminescence reagent (Pierce, Rockford, IL, USA). ChemiDoc Touch Imaging System with Image Lab Touch Software (BioRad) was used for image acquisition and densitometry analysis. Densitometry data are presented as ratio of target protein to Hsp90.

### Statistical Analysis

Graph Pad Prism 9.1 software was used for all graphical data presentation and statistical analysis. Data are presented as scatter plot (correlation analysis and categorical analysis, with median line). Details for statistical analysis are indicated in the text or in figure legends as appropriate. Parasite density and percent Hz-bearing WBCs were log transformed prior to analyses. Binary analysis of non-normally distributed data of matched samples utilized the Wilcoxon matched-pairs signed rank test; unpaired analyses utilized the Mann-Whitney test. Correlation analysis was performed using the Spearman’s correlation test. Multiple group comparisons were performed with Kruskal-Wallis test with post-hoc group-wise comparisons by Dunn’s multiple comparisons test. Two-tailed Fisher’s exact test was used to compare proportions. Statistical significance was set at *P <* 0.05.

## Results

### Granulocyte Counts Are Differentially Impacted by Malaria and HIV Infection in the Peripheral and Placental Blood

**Figure 2:**
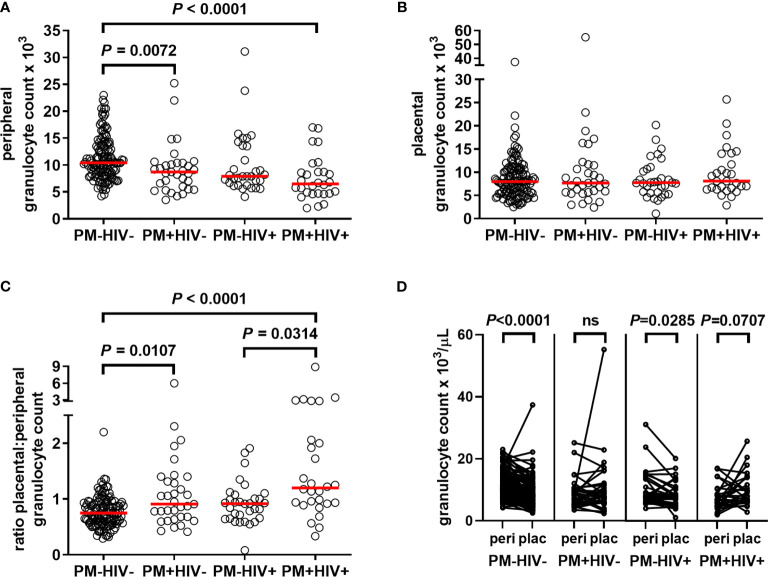
Placental malaria and HIV infections differentially alter granulocyte levels in peripheral and placental blood. Peripheral and placental blood were subjected to complete blood count. Granulocyte numbers in **(A)** peripheral and **(B)** placental blood are shown. **(C)** Depicts the ratio of placental to peripheral blood granulocyte numbers, HIV-/PM-, n = 131; HIV-/PM+, n = 33; HIV+/PM-, n = 32; HIV+/PM+, n = 28. **(A–C)** Statistics by Kruskal-Wallis test with post-hoc group-wise comparisons by Dunn’s multiple comparisons test. **(D)**, pairwise comparisons by Wilcoxon matched-pairs signed rank test. PM-=placental malaria negative; PM+=placental malaria positive; HIV-=human immunodeficiency virus seronegative; HIV+=human immunodeficiency virus seropositive.

To determine the extent to which granulocyte levels are influenced by PM, and how pre-existing HIV infection may modify this response, granulocyte counts derived from CBC analysis of peripheral and placental blood from women of all gravidities were assessed. Counts do not vary with gravidity in peripheral (median, IQR: paucigravid, 10.0 (6.90 – 13.7) x10^3^/μL; multigravid, 9.10 (7.30 – 11.5) x10^3^/μL; *P* = 0.6543, Mann Whitney test) or placental blood (paucigravid, 7.80 (5.70 – 11.9) x10^3^/μL; multigravid, 7.80 (5.70 – 10.4); *P* = 0.4889). Compared with control PM-HIV- women, peripheral granulocyte counts are reduced in PM+HIV- and PM+HIV+ women (**Figure 2A**). The lower levels in the latter are strongly attributable to HIV infection, as levels between the two HIV+ groups are not significantly different (*P* = 0.5044). In contrast to peripheral blood, no differences in neutrophil counts in the placenta are evident (**Figure 2B**). Interestingly, the ratio of placental to peripheral blood granulocyte counts is enhanced by PM in both HIV- and HIV+ women (**Figure 2C**). Pairwise comparison of peripheral to placental granulocyte counts among individuals within the infection groups shows that while placental granulocyte levels are reduced relative to the periphery in uninfected and HIV seronegative women, this difference is lost with PM, with a tendency toward a reversed pattern in co-infected women (**Figure 2D** and **Supplemental Table 1**). Additionally, with the exception of PM+HIV+ women, in whom total WBC counts are strongly elevated in placental relative to peripheral blood, relative patterns of granulocyte levels between the peripheral and placental blood vary independently of total WBC counts (**Supplemental Table 1** and **Supplemental Figure 2A**). In terms of proportion of total WBCs, granulocytes are universally substantially reduced in the placenta relative to the peripheral blood (**Supplemental Figure 2B** and **Supplemental Table 1**). However, whereas granulocyte percentages in the peripheral blood (**Supplemental Figure 2C**) follow the same trends observed with granulocyte counts (**Figure 2A**), placental granulocyte percentages reveal a distinct pattern of reduction in HIV+PM+ women (**Supplemental Figure 2D**) that is not observed in placental granulocyte counts (**Figure 2B**).

### Granulocyte Counts Correlate With Indicators of PM Severity but Not Birth Outcomes

**Figure 3:**
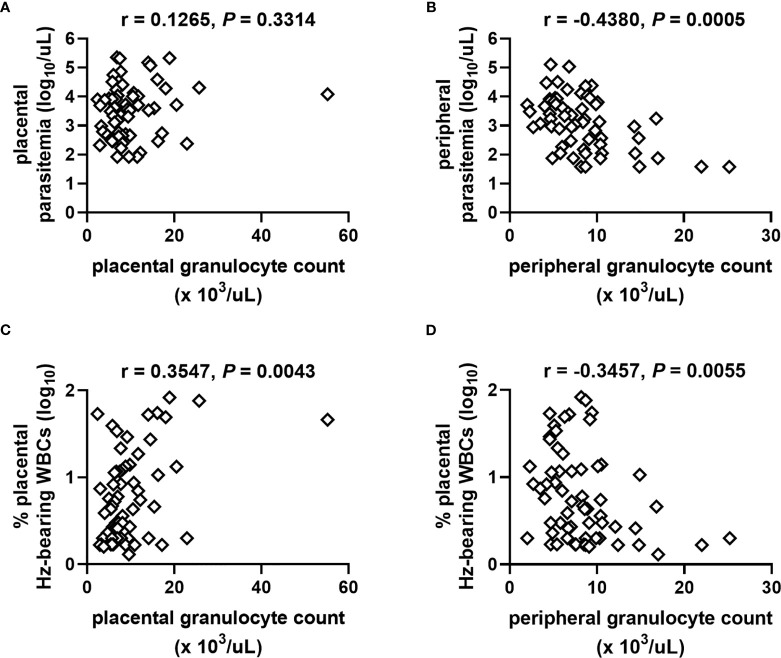
Relationships of granulocyte numbers with measures of placental malaria vary as a function of blood source. Placental granulocyte counts measured by complete blood count in women with PM are unrelated to **(A)** placental parasite density but positively correlate with **(C)** the level of Hz-bearing WBCs in the placental intervillous space. Peripheral granulocyte counts negatively correlate with **(B)** peripheral parasite density and **(D)** level of Hz-bearing WBCs in the placental intervillous space. Both infection measures are log transformed. Results of Spearman’s correlation analysis are shown. **(A, B)** n = 61; **(C, D)** n = 63.

PM often manifests as a chronic maternal inflammatory response dominated by accumulation of monocytes (5, 20, 26, 69). To assess the extent to which granulocyte counts might be influenced by severity of PM, correlation analysis of counts among PM+ women (combined HIV- and HIV+) were performed with parasite density and the percentage of WBCs bearing Hz in the placental blood space, which is taken as an indicator of chronicity of placental infection. The placental granulocyte count is unrelated to placental parasite density (**Figure 3A**) but positively correlates with Hz-bearing WBCs (**Figure 3C**). In contrast, the peripheral granulocyte count is inversely related to peripheral parasite density (**Figure 3B**) and Hz-bearing WBCs in the placenta (**Figure 3D**).

While granulocyte levels appear to be influenced by the intensity of the PM infection, no relationship with infant birth weight or gestational age is evident (**Supplemental Table 2**) nor do counts vary as a function of maternal self-reported fever (data not shown).

### Placental Blood MPO Increases With Placental Inflammation

**Figure 4:**
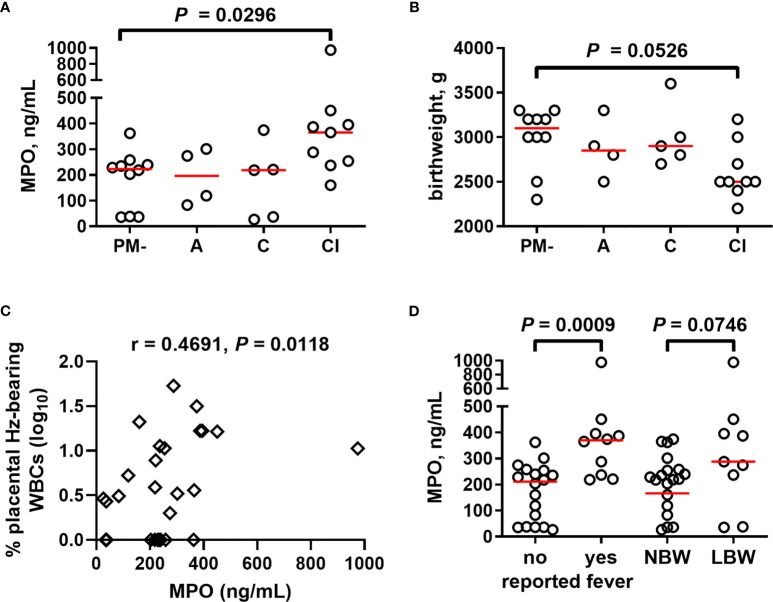
Placental blood MPO levels increase in chronic, inflammatory placental malaria, together with reduced birth weight. **(A)** MPO measured in placental plasma by ELISA from primigravid women with and without PM stratified by placental histopathological status (see Methods). **(B)** Infant birth weight from the same women is similarly stratified. **(C)** MPO levels collectively analyzed by Spearman’s correlation test with percent placental leukocytes (WBCs) bearing engulfed hemozoin. **(D)** Placental MPO levels measured by ELISA assessed for relationships with self-reported fever and infant birth weight. Statistics by Kruskal-Wallis test with post-hoc group-wise comparisons by Dunn’s multiple comparisons test. PM- = placental malaria negative (n = 10); A = acute infection (n = 4); C = chronic infection (n = 5); CI = chronic inflammatory infection (n = 9); NBW= normal birth weight (n = 19); LBW = low birth weight (n = 9); no self-reported fever (n = 18), reported fever (n = 10).

Based upon parameters including presence of parasites, inflammatory cell infiltration, and presence of Hz, PM has been variously categorized in histopathological examination in attempts to summarize severity and longevity of PM and associated birth outcomes (70–73). As a first step toward probing functional attributes of neutrophils in PM, a pilot study was conducted to measure levels of MPO in placental plasma from HIV-seronegative primigravidae whose placentae were histologically categorized into four groups (uninfected, acute, chronic, and chronic, inflammatory infection). MPO is released upon activation of neutrophils in the blood and tissues into both the phagolysosomal compartment and the extracellular environment, as well as in NETs (74, 75). Relative to uninfected samples, placental plasma from tissues with evidence of chronic, inflammatory infection shows significantly elevated MPO levels (**Figure 4A**). Correspondingly, this group tends toward lower infant birth weights relative to uninfected women (**Figure 4B**). Placental MPO levels correlate positively with percent of Hz-bearing WBCs in the placenta (**Figure 4C**). MPO levels are elevated with self-reported fever in these women and tend toward higher levels in those delivering low birth weight infants (**Figure 4D**). Overall, these data indicate that high MPO concentrations are associated with PM, especially chronic, inflammatory infection, and relevant clinical outcomes.

### Malaria and HIV Infection Impact Placental but Not Peripheral Levels of Soluble Markers of Neutrophil Activation

**Figure 5:**
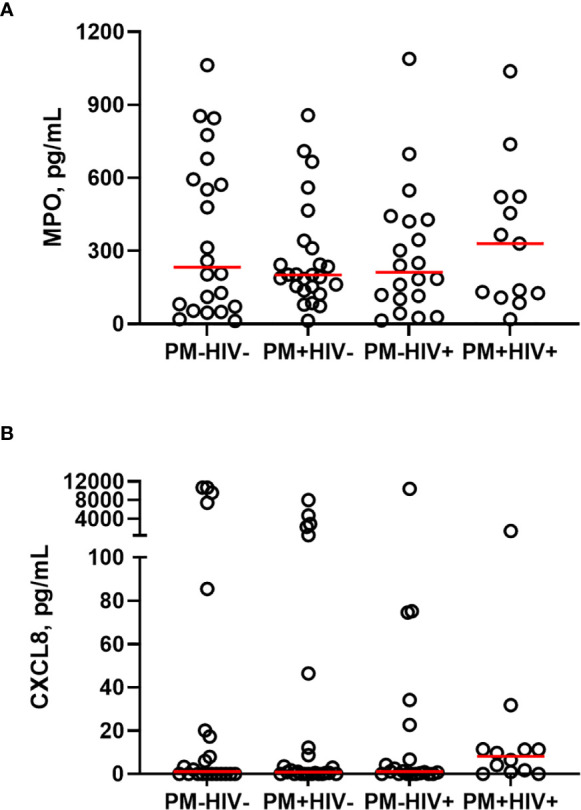
Peripheral blood MPO and CXCL8 levels do not change as a function of placental malaria and HIV seropositivity. Peripheral plasma samples from paucigravid women were analyzed for **(A)** MPO and **(B)** CXCL8 levels by bead array. No statistically significant differences among the groups are evident (Kruskal-Wallis test with post-hoc group-wise comparisons by Dunn’s multiple comparisons test). HIV-/PM-, n = 22; HIV-/PM+, n = 25; HIV+/PM-, n = 20; HIV+/PM+, n = 14 (MPO), n = 13 (CXCL8.) Groups as defined in **Figure 2**.

**Figure 6:**
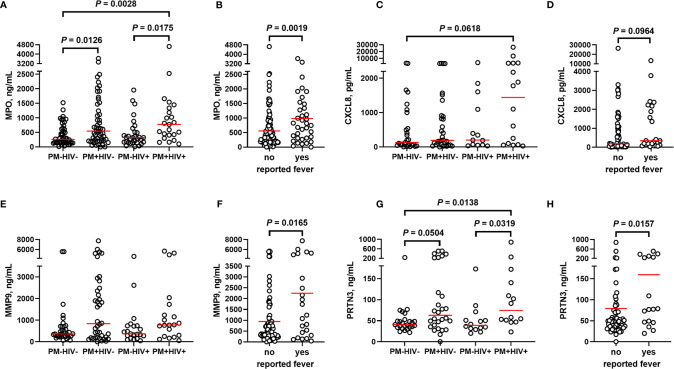
Markers of neutrophil activation are variably altered by placental malaria and HIV seropositivity. Placental plasma from paucigravid women was analyzed for **(A, B)** myeloperoxidase (MPO), **(C, D)** CXCL8, **(E, F)** matrix metalloproteinase 9 (MMP9), and **(G, H)** proteinase 3 (PRTN3) levels by bead array and ELISA and stratified by **(A, C, E, G)** infection status and **(B, D, F, H)** self-reported fever (independent of infection status). **(A, C, E, G)** Statistics by Kruskal-Wallis test with post-hoc group-wise comparisons by Dunn’s multiple comparisons test and **(B, D, F, H)** by Mann-Whitney U test. MPO: HIV-/PM-, n = 50; HIV-/PM+, n = 53; HIV+/PM-, n = 31; HIV+/PM+, n = 24. CXCL8, HIV-/PM-, n = 27; HIV-/PM+, n = 33; HIV+/PM-, n = 13; HIV+/PM+, n = 16. MMP9, HIV-/PM-, n = 35; HIV-/PM+, n = 40; HIV+/PM-, n = 21; HIV+/PM+, n = 21. PRTN3, HIV-/PM-, n = 25; HIV-/PM+, n = 28; HIV+/PM-, n = 14; HIV+/PM+, n = 15. Groups as defined in **Figure 2**.

**Figure 7:**
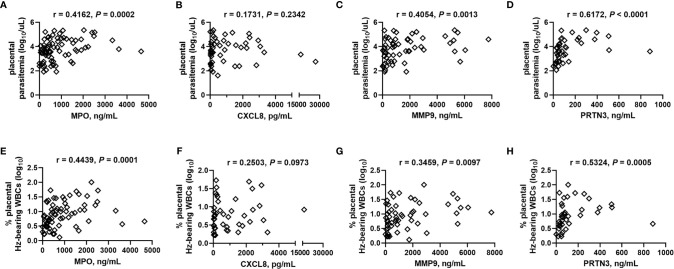
Placental blood MPO, MMP9, and PRTN3 levels positively correlate with placental parasite density and hemozoin-bearing white blood cells. **(A, E)** MPO, **(B, F)** CXCL8, **(C, G)** MMP9, and **(D, H)** PRTN3 levels measured by bead array in plasma are collectively analyzed by Spearman’s correlation test with **(A–D)** thick smear placental parasite density (log transformed) and **(E–H)** percent placental WBCs bearing engulfed hemozoin (log transformed). **(A)**, n = 77; **(B)**, n = 49; **(C)**, n = 61; **(D)**, n = 43; **(E)**, n = 73; **(F)**, n = 47; **(G)**, n = 57; **(H)**, n = 40.

Building upon these initial observations, a larger analysis was undertaken to identify the extent to which malaria and PM/HIV co-infections in pregnant women impact indicators of neutrophil function. In addition to MPO, key markers of activated neutrophils, MMP9, and PRTN3, and a key neutrophil chemoattractant, CXCL8, were measured in peripheral and placental plasma of paucigravid women. In peripheral blood, MPO and CXCL8 levels are not impacted by PM, regardless of HIV infection status (**Figures 5A, B**). Additionally, peripheral MPO and CXCL8 levels are unrelated to infant birth weight and gestational age at birth (**Supplemental Figures 3E, F**). In contrast, placental blood levels of MPO are significantly elevated with PM and PM/HIV co-infection relative to uninfected and HIV+ women, respectively (**Figure 6A**), and in mothers who reported recent fever (**Figure 6B**). CXCL8 levels in PM+HIV+ women are elevated relative to levels in uninfected women (**Figure 6C**), and tend toward a weak enhancement with reported fever (**Figure 6D**). While MMP9 levels remain unchanged with infection (**Figure 6E**), levels are enhanced with reported fever (**Figure 6F**). Finally, like MPO, PRTN3 levels are enhanced by PM in both HIV- and HIV+ women (**Figure 6G**), and are increased with recent fever (**Figure 6H**). Despite associations with PM, none of the measured markers differ as a function of birth weight or gestational age **(** **Supplemental Figure 3** **)**. Placental levels of all of these factors significantly positively correlate with each other (**Table 2**), and with the exception of CXCL8, all positively correlate with placental parasite density (**Figures 7A–D**), and percent Hz-bearing WBCs in the placenta (**Figures 7E–H**). However, contrary to expectation, none of these factors correlate with granulocyte counts or percentages (data not shown).

**Table 2:** Correlation matrix of placental markers of neutrophil activation.

	MPO		MMP9		PRTN3		CXCL8	
	r, IQR*	*P*	r, IQR	*P*	r, IQR	*P*	r, IQR	*P*
**MPO**	1	**-**	0.7287, 0.6273 - 0.8058	<0.0001	0.9179, 0.8737 - 0.9471	<0.0001	0.4485, 0.2002 - 0.6426	0.0006
**MMP9**	0.7287, 0.6273 - 0.8058	<0.0001	1	**-**	0.7626, 0.6500 - 0.8424	<0.0001	0.3797, 0.0804 - 0.6161	0.0120
**PRTN3**	0.9179, 0.8737 - 0.9471	<0.0001	0.7626, 0.6500 - 0.8424	<0.0001	1	**-**	0.4106, 0.0615 - 0.6701	0.0196
**CXCL8**	0.4485, 0.2002 - 0.6426	0.0006	0.3796, 0.0804 - 0.6161	0.0120	0.4106, 0.0615 - 0.6701	0.0196	1	**-**

*Data represent results of Spearman’s test with IQR, interquartile range.

### Placental MPO Detection by Immunohistochemistry and Western Blot

**Figure 8:**
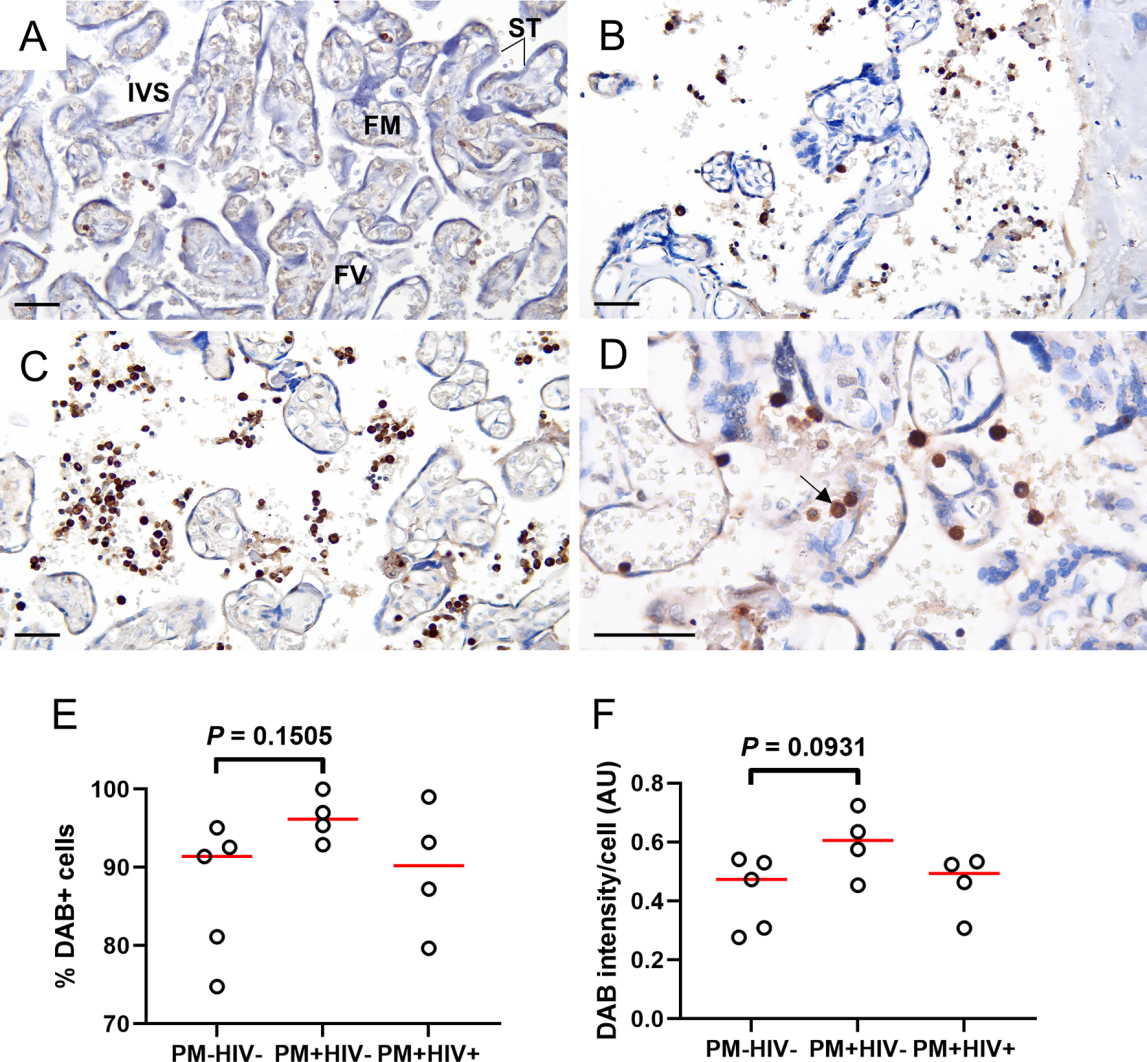
Placental WBCs from PM+HIV- women show more robust MPO expression by immunohistochemistry relative to uninfected and co-infected women. Frequency and intensity of MPO staining is lower in **(A)** uninfected placenta and **(B)** PM+HIV+ placenta relative to **(C)** PM+HIV- placenta. **(D)** Segmented nucleus clearly delineates MPO staining in a neutrophil (arrow). ST, syncytiotrophoblast; FM, fetal mesenchyme; FV, fetal vessel; IVS, intervillous space. Scale bars represent 50 μm. **(E)** Percent cells expressing MPO weakly tends to be higher in PM+HIV- relative to uninfected women. **(F)** MPO staining intensity tends to be higher in PM+HIV- relative to uninfected women. Statistics by Kruskal-Wallis test with post-hoc group-wise comparisons by Dunn’s multiple comparisons test. PM-/HIV-, n = 5; PM+/HIV-, n = 4; PM+/HIV+, n = 4. Groups as defined in **Figure 2**.

To further characterize MPO expression in the placenta, tissue sections from a subgroup of patients were probed using immunohistochemistry. MPO+ cells are frequently observed in these placentae, even in the absence of PM (**Figures 8A–D**). Frequency (**Figure 8E**) and intensity of MPO expression (**Figure 8F**), however, tend to be enhanced in PM+HIV- but not PM+HIV+ tissues. Probing for MPO by western blot of proteins derived from whole placental tissue may serve as a proxy for intensity of MPO expression, but preliminary analysis thereto does not reveal compelling evidence for influence of protein levels by infection status (**Supplemental Figures 4A, C**). Similarly, NE protein levels in whole placental tissue extracts are independent of infection status (**Supplemental Figure 4B, D** **)**.

### Plasma Markers of NETosis Are Not Affected by PM

Although NETosis has been linked to severe malaria in nonpregnant patients (38, 56, 57), this mechanism has not to our knowledge been explored in PM. Using detection of nonspecific (cell-free DNA; **Supplemental Figure 5A**) and known markers of NET formation (NE-DNA, **Supplemental Figure 5B** **;** cell-free histones, **Supplemental Figure 5C**), no evidence of significant PM-induced NETosis in placental plasma is observed in HIV- primigravidae (as in **Figure 4**), regardless of placental histopathological status. Similarly, citrullinated histone H3 (citH3), an additional marker of NET formation, does not differ in paucigravid placental plasma as a function of PM regardless of HIV infection (**Supplemental Figure 6A**), although elevated levels are evident in two PM+ women. Nonetheless, citrullinated histone H3 levels tend to positively correlate with placental granulocyte count (**Supplemental Figure 6B**).

### *In Situ* Evidence for Netosis in Placental Tissue

As an alternate indicator of NETosis, fixed placental tissue was probed by immunohistochemistry for citH3. Some weakly positive cells are evident in the intervillous space, and, surprisingly, occasional stronger staining is seen in syncytiotrophoblast (**Supplemental Figure 7A, B** **)**. Extracellular evidence of citH3, structures that would be consistent with NETosis, however, is rarely observed (**Supplemental Figure 4B**).

**Figure 9:**
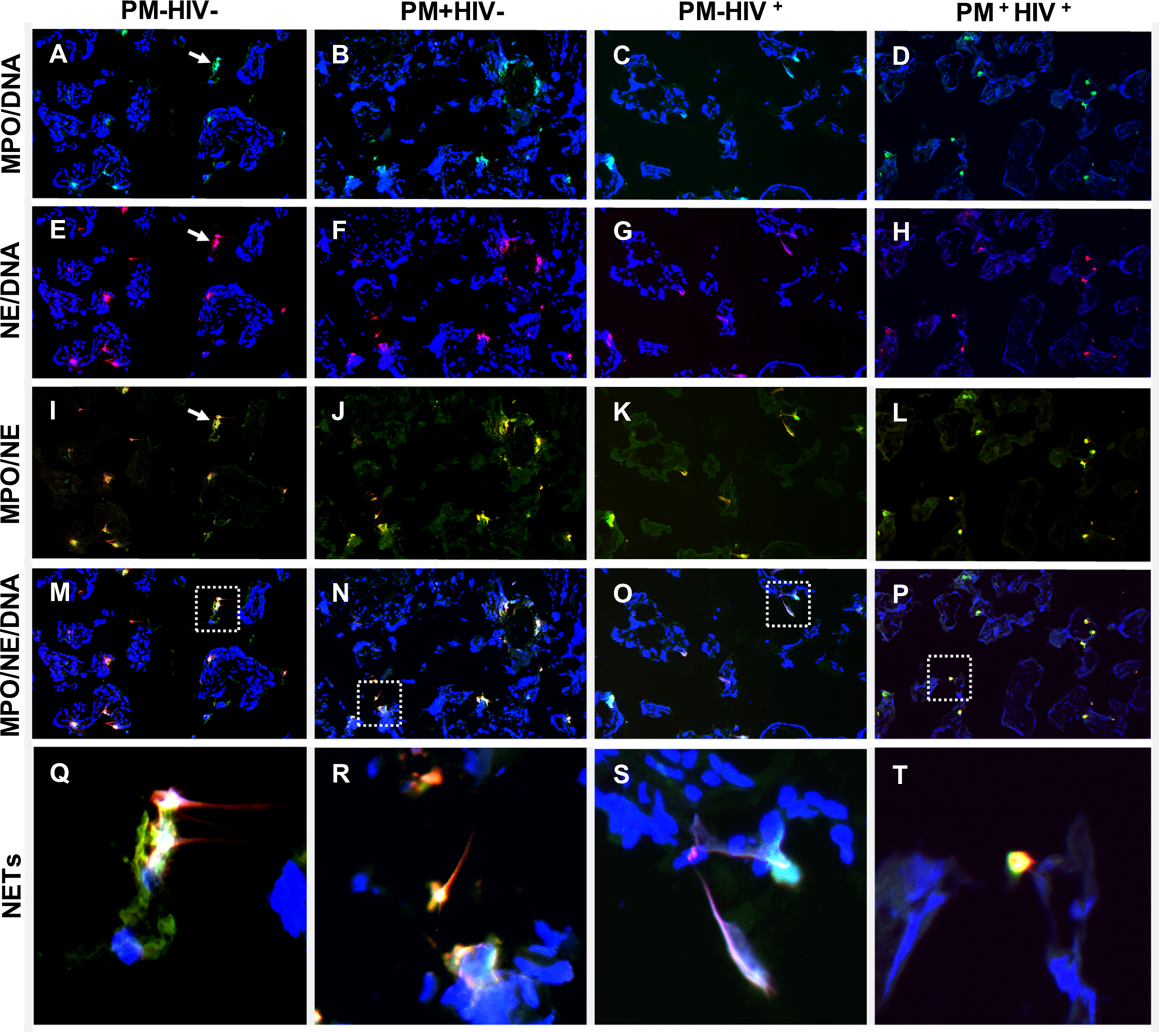
Detection of NETs by immunfluorescence in placental tissue. Immunolocalization of MPO (green), NE (red) in term placenta. The DNA was labeled with Hoescht 33342 (blue). **(A, E, I, M, Q)** uninfected placenta; **(B, F, J, N, R)** PM+HIV- placenta; **(C, G, K, O, S)** PM-HIV+ placenta; and **(D, H, L, P, T)** PM+HIV+ placenta. The first row **(A–D)** shows the colocalization of MPO with DNA, the second row **(E–H)**, colocalization of NE and DNA, the third row **(I–L)**, colocalization of MPO and NE and the fourth row **(M–P)**, colocalization of MPO, NE and DNA. The last row shows in higher magnification the dotted areas in panels **(M–P)** that contain putative NET-like structures **(Q–T)**. Arrows in panels **(A, E, I)** indicate NET-like structures. Groups are as defined in **Figure 2**.

Although attempts to identify soluble components of NETosis in placental plasma and *in situ* in fixed, paraffin-embedded placental tissue does not provide compelling evidence of PM with or without HIV co-infection as a driver of placental NETosis, it is not possible to conclude that this process does not occur in PM, since sample collection, processing and storage, for example, could impede such detection. As an alternate approach, placental tissues flash-frozen in OCT were assessed by immunofluorescence staining for MPO, NE and DNA. In most samples, regardless of infection status, structures that are suggestive of NETs are evident (**Figure 9** **;** **Supplemental Figure 8**). The presumptive NETs are highly pleomorphic. Some structures are composed of single cells in the intervillous space that have streaky, comet-like nuclei and cytoplasm containing blue fluorescence (DNA) that colocalizes with green fluorescence (MPO) and red fluorescence (NE) (**Figures 9M–P, R–T**). Most frequently, the NETs are characterized by small to large aggregates of two or more pleomorphic cells with indistinct cell limits and pleomorphic nuclei encroached in a large MPO+/NE+ cytoplasm (**Figures 9M–O, Q–S**). Frequently, the cytoplasmic projections colocalize with MPO, NE and DNA material (**Figures 9M–O, Q–S**). These presumptive NETs are observed in maternal intervillous blood spaces, within the villus stroma, and occasionally inside the fetal vasculature (**Figures 9S, T**).

**Figure 10:**
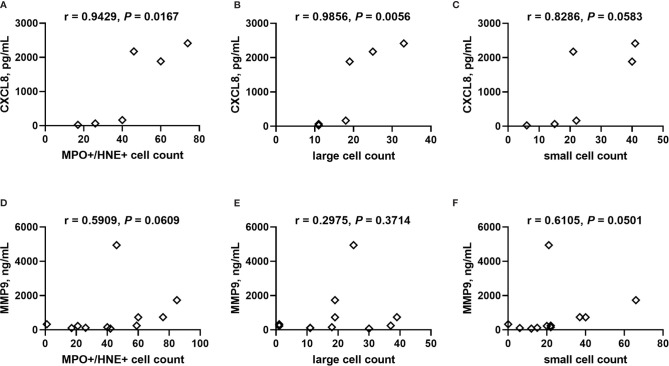
Correlation analysis of CXCL8 and MMP9 with neutrophils in placental tissue. Counts of neutrophils detected in frozen cryosections as **(A, D)** MPO+/NE+, and MPO+/NE+ cells stratified by size as **(B, E)** large: >102 μm^2^, and **(C, F)** small: <102 μm^2^ collectively correlated with placental plasma levels of **(A–C)** CXCL8 and **(D–F)** MMP9. Cell counts determined by analysis of immunofluorescence of tissues. Statistics by Spearman’s correlation test. PM-/HIV-, n = 5; PM+/HIV-, n = 5; PM-/HIV+, n = 4; PM+/HIV+, n = 4. Groups as defined in **Figure 2**.

The small sample size of this experiment impacts statistical power; however, counts of singly stained MPO+, NE+ and MPO+/NE+ double stained cells do not vary with PM, including with stratification by HIV serostatus (**Supplemental Figures 8A–C**). Small, non-NETosing neutrophils (double MPO+/NE+, <102 μm^2^) tend to be more numerous with PM (median, IQR, PM-: 12, 4.5 – 60; PM+: 22, 18 – 41; *P* = 0.0928) whereas larger putatively NETosing MPO+/NE+ cells are unchanged (median, IQR, PM-: 9.0, 2.0 – 35; PM+: 19, 15 – 34; *P* = 0.3735). Further stratification by HIV infection status yields no significant differences between the groups for small and large cells, including assessment of large NETosing cells as a percent of all MPO+/NE+ cells (**Supplemental Figures 8D–F**). Counts of MPO+/NE+ cells, including those classified as large, positively correlate with placental levels of CXCL8 (**Figures 10A, B**), with a similar tendency for small MPO+/NE+ neutrophils (**Figure 10C**). Likewise, weak positive correlations are observed between MMP9 and MPO+/NE+ cells as well as small neutrophils (**Figures 10D, F**) but not between MMP9 and large cell count (**Figure 10E**). Placental levels of MPO are unrelated to these cell counts (**Supplemental Figure 9**).

## Discussion

In this study, we analyzed maternal peripheral and placental granulocyte levels as well as markers of neutrophil activation, including NET-specific markers and structures, in women with and without PM and HIV infection at the time of delivery. The results show that maternal peripheral granulocyte counts decrease with PM in the presence and absence of pre-existing HIV infection. Although a differential to discern neutrophil counts among the granulocytes was not performed, these findings are consistent with previous work that reported reduced neutrophil counts in malaria-infected pregnant compared to uninfected women (3, 76). A study in rhesus monkeys (*Macaca mulatta*) infected with *P. coatneyi* also showed a significant decrease in neutrophil levels starting at gestation week 9 (77). This contrasts with the observation that neutrophil counts are enhanced with HIV infection in pregnant women (78). Our results suggest that PM overcomes this apparent HIV-driven neutrophilia, since HIV seropositive women with PM have significantly suppressed granulocyte counts in the peripheral blood.

Contrary to expectation, no differences in granulocyte counts in the placenta are evident in this cohort of women. However, the enhanced ratios of placental to peripheral blood granulocyte numbers at the population level support the conclusion that during PM and PM/HIV co-infection, granulocytes accumulate in the placenta at the expense of the periphery, or, at minimum, are relatively more stable in the placenta. The physiological control of this phenomenon is worthy of further investigation, particularly given our observation that overall, granulocytes make up a lower proportion of total leukocytes in the placenta than in the peripheral blood and thus may be differentially regulated in these two blood spaces. Neutrophils are a key component of the normal process of labor, with accumulation of these cells in the uterine wall; the reductions observed here may be indicative of an exodus of neutrophils from the intervillous space to the myometrium [reviewed in (79)]. Despite the tendency toward lower granulocyte percentages and counts in placental relative to peripheral blood in the absence of infection, pairwise comparison at the individual patient level shows an overall tendency for granulocyte counts in placenta to increase uniquely with PM/HIV co-infection. However, granulocytes as a percent of total WBCs in the placenta are significantly reduced with co-infection. This may indicate that the massive overall increase in placental WBCs seen in this group (**Supplemental Table 1**) is heavily attributable to other cell subsets, likely monocytes (20).

Other researchers have reported significant accumulation of neutrophils in the placenta with PM (19, 20). What may be driving accumulation or preservation of these cells in the maternal placental blood, and why the current study shows placental granulocyte count stability but not accumulation with PM is unclear. Aside from the obvious differences in methodology and cell identification, placental granulocyte levels as measured here appear to be influenced by infection intensity, as counts positively correlate with the percent of placental WBCs bearing phagocytosed Hz. Thus, differences across studies could also be due to differences in infection intensity or chronicity. In general, local production of chemokines, including CXCL8, a factor that promotes neutrophil chemotaxis (80), may be an important determining factor in granulocyte/neutrophil presence in the intervillous blood, as is the case for recruitment to the uterus at parturition [reviewed in (79)]. Importantly, CXCL8 is elevated in PM+HIV+ placentae. The source of CXCL8 in the placenta that might participate in this response is not clear but could be maternal monocytes (22, 26), fetal syncytiotrophoblast (3, 22, 81, 82), or uterine/decidual stromal cells (83).

Previous studies have indicated that neutrophils may play a pathogenic role in PM and could serve as prognostic markers for malaria-associated low birth weight (18). We report that in this population of parturient women, granulocyte counts do not associate with infant birth outcomes. This may be due to inadequate sample size, lack of differential analysis to directly count neutrophils, or may be related to the overall patient recruitment strategy, which excluded complicated pregnancies and deliveries and health issues other than malaria and HIV infections. Because an interesting relationship between neutrophils and malaria parasites is emerging [reviewed in (36)] and neutrophils are key cells at multiple stages of normal and abnormal pregnancy [reviewed in (79)], it is imperative for future studies to definitively identify neutrophils in the granulocyte population, and to further consider neutrophil subsets and functional parameters to discern potential relationships with pathogenic outcomes of PM.

One of the many consequences of neutrophil activation is the secretion of granules containing MPO, MMP9, NE, and PRTN3. Neutrophils produce massive amounts of these proteins, in the case of MPO, representing 5% of the total cellular protein (84). Our data show for the first time that placental blood MPO and PRTN3 levels are elevated with PM in both HIV- and HIV+ paucigravid women relative to PM-HIV- women. It was unexpected to find that none of these markers correlates with placental granulocyte counts, emphasizing the need for further work to definitively identify the source of these factors in placental plasma and fully characterize neutrophil function in PM. Importantly, observations of MPO in fixed placental tissue sections suggest that this factor may be produced by both intervillous neutrophils and monocytes, yet most MPO-expressing cells observed by immunofluorescence are co-stained for NE, identifying them as neutrophils.

Placental levels of MPO, MMP9, and PRTN3 all positively correlate with placental parasitemia as well as placental hemozoin bearing WBCs, suggesting that production is enhanced by chronic infection. Since MPO attenuates pathogen clearance during *P. yoelii* nonlethal infection (85), it is tempting to speculate that this enzyme may inhibit parasite clearance in PM as well. In an initial pilot study, we found placental blood MPO levels to be increased significantly with chronic, inflammatory PM in primigravidae. MPO is associated with vascular dysfunction (86) and underlies the pathophysiology of numerous vascular inflammatory diseases including arteriosclerosis and coronary artery disease (87, 88). Inflammation and endothelial dysfunction are characteristics of preeclampsia, and increased MPO levels in placental and peripheral circulation in preeclamptic women have been described (89, 90). The extent to which PM and PM/HIV coinfection may contribute to preeclampsia *via* MPO production or modification of other neutrophil functions remains to be determined. This may be an exciting avenue to pursue given increasing evidence that malaria predisposes women to this hypertensive disorder (91, 92).

Elevated neutrophil activation, as evidenced by higher plasma concentrations not only of MPO but also of PRTN3 and NE, is associated with severe pediatric malaria (39). Likewise, MMP9, an endopeptidase released by neutrophils and monocytes, is implicated in the pathogenesis of severe malaria (93–95). An MMP9 polymorphism protects against PM, further implicating an important role for this enzyme in *P. falciparum* infection (96). Importantly, MPO, MMP9 and PRTN3 levels in the placenta associate with self-reported fever in this cohort of parturient women. However, while pilot data in HIV seronegative primigravidae suggest a tendency for placental MPO to be higher in women with low birth weight infants, in a larger cohort of paucigravid malaria-exposed women that also considered HIV infection, no relationship with birth weight or gestational age emerges for any of these markers. This is unexpected given that the factors do correlate with parameters (placental parasite density and Hz-bearing WBCs) that are typically associated with poor outcomes in PM. Indeed, Hz-bearing neutrophils in the peripheral blood of pregnant women predict low birth weight (18). While additional work will be required to resolve this discrepancy, these results suggest that the mechanism by which neutrophils mediate poor birth outcomes may not be directly related to the release of neutrophil granules in the placental blood space. One potential alternate mechanism may be downstream effects of these factors, which were not measured here. High concentrations of neutrophil-derived antimicrobial compounds can make these cells detrimental to the host (97–99). For example, activated neutrophils exacerbate preeclampsia by releasing ROS (100–105). Of note, MPO catalyzes the formation of aggressive reactive oxygen intermediates, including hypochlorous, hypobromous, and hypothiocyanous acids, respectively (106, 107), which contribute to oxidative killing (108). Oxidative stress is a feature of PM (43, 44), but the extent to which neutrophils contribute to it remains to be established.

Contrary to expectation, this study finds no evidence of a relationship between PM and levels of soluble markers of NETosis in the placenta. Similarly, while structures consistent with NETs (MPO, NE, and DNA co-localization) are observed in the intervillous space, a relationship with PM is not evident. Alternatively, non-activated, resting, “small” neutrophils tend to be elevated with PM in this study. Because NETs have been observed in the placental intervillous space in pregnancies complicated by preeclampsia (103, 105), and given parallels between PM and preeclampsia, it was our expectation that NETosis would be enhanced in placentae of infected women. While further research designed specifically to address this question is warranted, it is tempting to speculate that NETosis may be subject to unique and as yet poorly understood control mechanisms in the placenta that are not specifically activated or perturbed by PM. The overall reduction of granulocyte counts in the placenta relative to the peripheral blood hints at this possibility.

In conclusion, the present study demonstrates that PM and PM/HIV co-infection perturb granulocyte levels, and soluble signatures of neutrophil activation associate with indicators of PM infection and associated symptoms. The findings do not authoritatively distinguish between a protective or pathogenic role for neutrophils or products of their activation, nor is an association of PM with NETosis established. Further exploration of neutrophil function in the context of malaria and HIV in pregnant women, particularly direct assessment of activity, is required to fill remaining gaps in knowledge.

## Data Availability Statement

The original contributions presented in the study are included in the article/**Supplementary Material**. Further inquiries can be directed to the corresponding author.

## Ethics Statement

The study supporting collection of samples used in this report was approved by the Kenya Medical Research Institute Ethical Review Committee, and the Centers for Disease Control and Prevention and the University of Georgia Institutional Review Boards. All study participants provided written informed consent before enrollment and procedures and instruments involving human subjects, sample collection and data analysis, processing, and testing were approved throughout the conduct of patient recruitment. All samples and data are anonymized.

## Author Contributions

JMM, LJO, BR, and DS conceived and designed the experiments. LA, JDM, JMM, SM, and SO coordinated sample and clinical data collection. FA, JMM, LJO, SO, BR, BNR, and DS performed the experiments. LJO performed the Qupath analysis and prepared micrographs. JMM, LJO, BNR, and DS prepared the figures and tables. JMM performed descriptive statistical analyses. JMM, LJO, and DS wrote the manuscript. JV provided logistical and infrastructural support for sample collection, processing, storage and shipment, and facilitated local ethical reviews in Kenya. All authors (with the exception of JV, deceased) contributed to the article and approved the submitted version.

## Funding

This work was supported by the National Institutes of Health grants R01 AI050240 and R21 AI111242, and research support from the University of Florida to JMM. The content is solely the responsibility of the authors and does not necessarily represent the official views of the National Institute of Allergy and Infectious Diseases (NIAID) or the National Institutes of Health. The funders had no role in study design, data collection and analysis, decision to publish, or preparation of the manuscript.

## Conflict of Interest

LA was employed by #1 Heartsaved Adult Family Care.

The remaining authors declare that the research was conducted in the absence of any commercial or financial relationships that could be construed as a potential conflict of interest.

## Publisher’s Note

All claims expressed in this article are solely those of the authors and do not necessarily represent those of their affiliated organizations, or those of the publisher, the editors and the reviewers. Any product that may be evaluated in this article, or claim that may be made by its manufacturer, is not guaranteed or endorsed by the publisher.

## Supplementary Material

Supplementary Table 1 Pairwise comparison of peripheral and placental white blood cell (WBC) and granulocyte counts. PM = placental malaria; HIV = human immunodeficiency virus. Data are shown in graphical form in **Supplemental Figure 2A**, and **Supplemental Figure 2B**.

Supplementary Table 2 Granulocyte numbers as a function of infant birth weight and gestational age at birth. NBW, normal birth weight, LBW, low birth weight; TB, term birth; PTB, preterm birth.
