# Supplementary material for: Myeloperoxidase and Other Markers of Neutrophil Activation Associate With Malaria and Malaria/HIV Coinfection in the Human Placenta
Source: Front Immunol. 2021 Oct 19;12:682668. doi: 10.3389/fimmu.2021.682668 (PMC8562302; doi:10.3389/fimmu.2021.682668)
Supplement: Supplementary Table 1 — Pairwise comparison of peripheral and placental white blood cell (WBC) and granulocyte counts. PM = placental malaria; HIV = human immunodeficiency virus. Data are shown in graphical form in Supplemental Figure 2A , and Supplemental Figure 2B . [file Table_1.pdf]

Supplemental Table 1: Pairwise comparison of peripheral and placental white blood cell (WBC) and granulocyte counts

		PM-HIV-	*P*	PM+ HIV-	*P*	PM-HIV+	*P*	PM+ HIV+	*P*
**Total WBC counts x $10^3/\mu L$ (median, interquartile range)**	**Peripheral**	13.1, 10.9-16.3	0.6207	11.4, 8.25-13.2	0.0024	10.5, 8.60-16.1	0.0762	10.4, 7.05-12.1	<0.0001
	**Placental**	13.7, 10.9-16.6		13.0, 10.5-20.0		12.3, 10.6-16.2		16.7, 13.2-23.4	
**Total granulocyte counts x $10^3/\mu L$ (median, interquartile range)**	**Peripheral**	10.4, 8.30-13.7	<0.0001	8.70, 5.75-10.4	0.4607	7.90, 6.33-13.6	0.0285	6.50, 4.70-8.70	0.0707
	**Placental**	8.00, 5.70-10.6		7.70, 5.70-11.6		7.75, 5.55-10.6		8.10, 6.38-13.4	
**Granulocyte percent of total WBC (mean ± SD)**	**Peripheral**	79.4 ± 8.30	<0.0001	76.8 ± 6.80	<0.0001	78.8 ± 8.20	<0.0001	68.7 ± 14.5	<0.0001
	**Placental**	57.7 ± 13.5		57.1 ± 10.0		59.4 ± 10.1		50.7 ± 14.2	

PM = placental malaria; HIV = human immunodeficiency virus. Data are shown in graphical form in Supplemental figure 2A, Figure 2D, and Supplemental figure 2b.
