# Supplementary material for: Myeloperoxidase and Other Markers of Neutrophil Activation Associate With Malaria and Malaria/HIV Coinfection in the Human Placenta
Source: Front Immunol. 2021 Oct 19;12:682668. doi: 10.3389/fimmu.2021.682668 (PMC8562302; doi:10.3389/fimmu.2021.682668)
Supplement: Supplementary Table 2 — Granulocyte numbers as a function of infant birth weight and gestational age at birth. NBW, normal birth weight, LBW, low birth weight; TB, term birth; PTB, preterm birth. [file Table_2.pdf]

Supplemental Table 2: Granulocyte numbers as a function of infant birth weight and gestational age at birth.

		**Birth weight**			**Gestational age**		
		**NBW (>2500 g)** (n=205)	**LBW (≤2500 g)** (n=19)	***P***	**TB (≥37 weeks)** (n=205)	**PTB (<37 weeks)** (n=19)	***P***
**Total granulocyte counts x $10^3/\mu L$ (median, interquartile range)**	**Peripheral**	9.30, 7.15-12.4	9.90, 6.20-17.0	0.7894	9.40, 7.10-12.8	9.20, 6.10-13.1	0.6232
	**Placental**	7.80, 5.70-10.6	8.20, 5.40-13.9	0.5243	7.80, 5.70-10.6	8.20, 5.40-11.7	0.8335

NBW = normal birth weight, LBW = low birth weight, TB = term birth, PTB = preterm birth
