# Supplementary material for: Myeloperoxidase and Other Markers of Neutrophil Activation Associate With Malaria and Malaria/HIV Coinfection in the Human Placenta
Source: Front Immunol. 2021 Oct 19;12:682668. doi: 10.3389/fimmu.2021.682668 (PMC8562302; doi:10.3389/fimmu.2021.682668)
Supplement: Supplementary file 3 [file DataSheet_1.pdf]

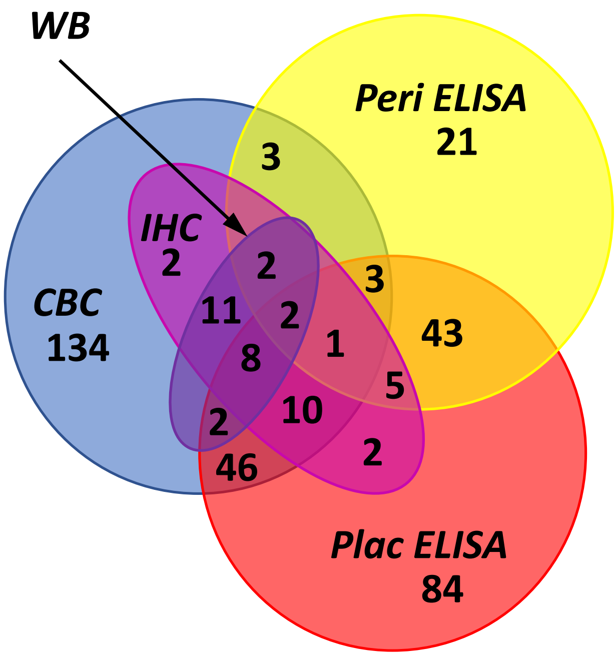

Supplemental figure 1. Venn diagram of data contribution per individual.
Refer to Figure 1 and text for details. Peri = peripheral plasma; plac = placental plasma; WB = western blot; IHC = immunohistochemistry and immunofluorescence; CBC = complete blood count.

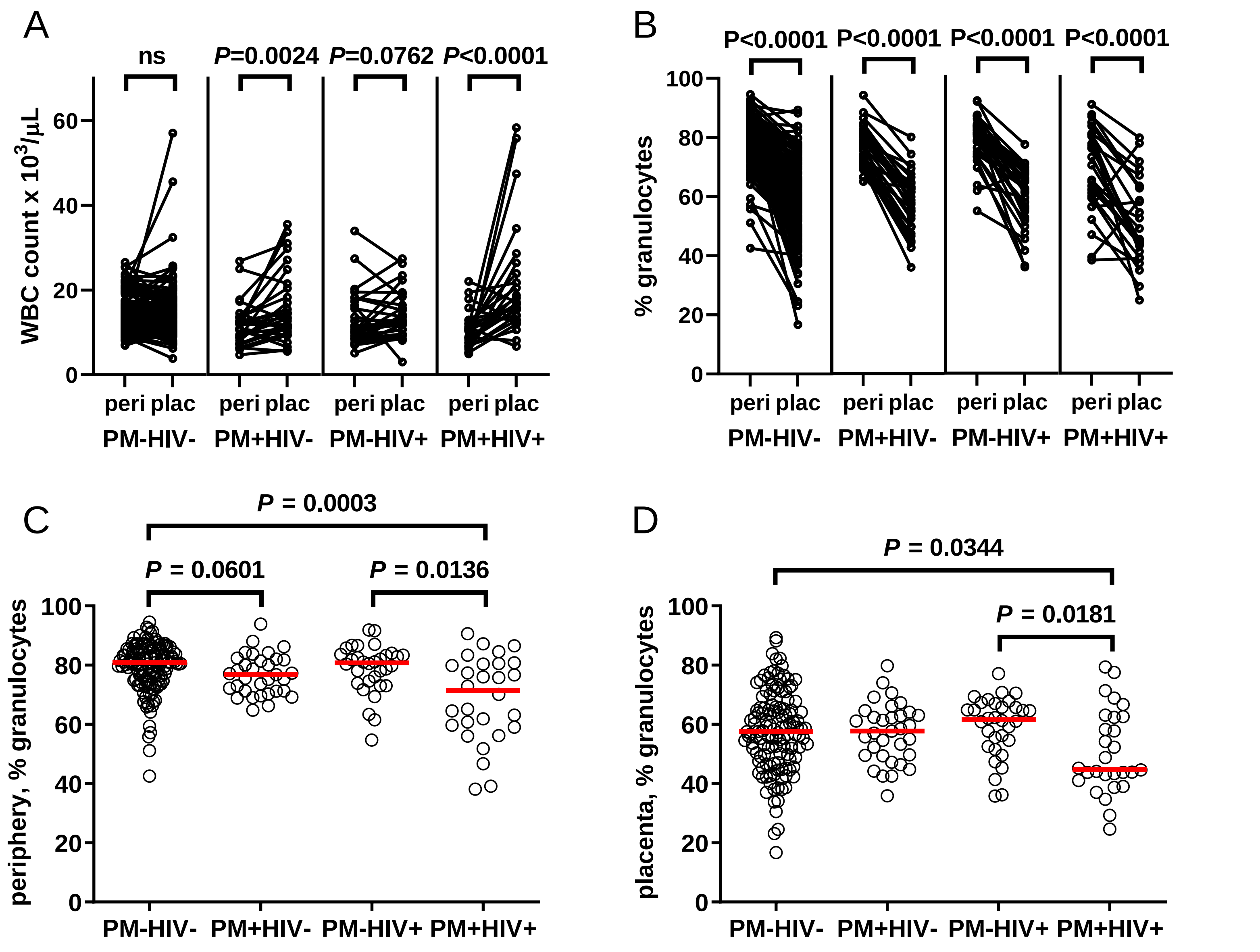

Supplemental figure 2. Total white blood cell (WBC) counts (A) and granulocytes as a percent of total WBCs (B-D) in peripheral and placental blood.
Data are stratified by infection status and A,B) pairwise comparisons performed with Mann Whitney U test between peripheral and placental blood are shown. C,D) Granulocytes as a percent of total WBCs, compared by Kruskal Wallis test with post-hoc group-wise comparisons by Dunn's multiple comparisons test. Samples sizes as in Figure 1.

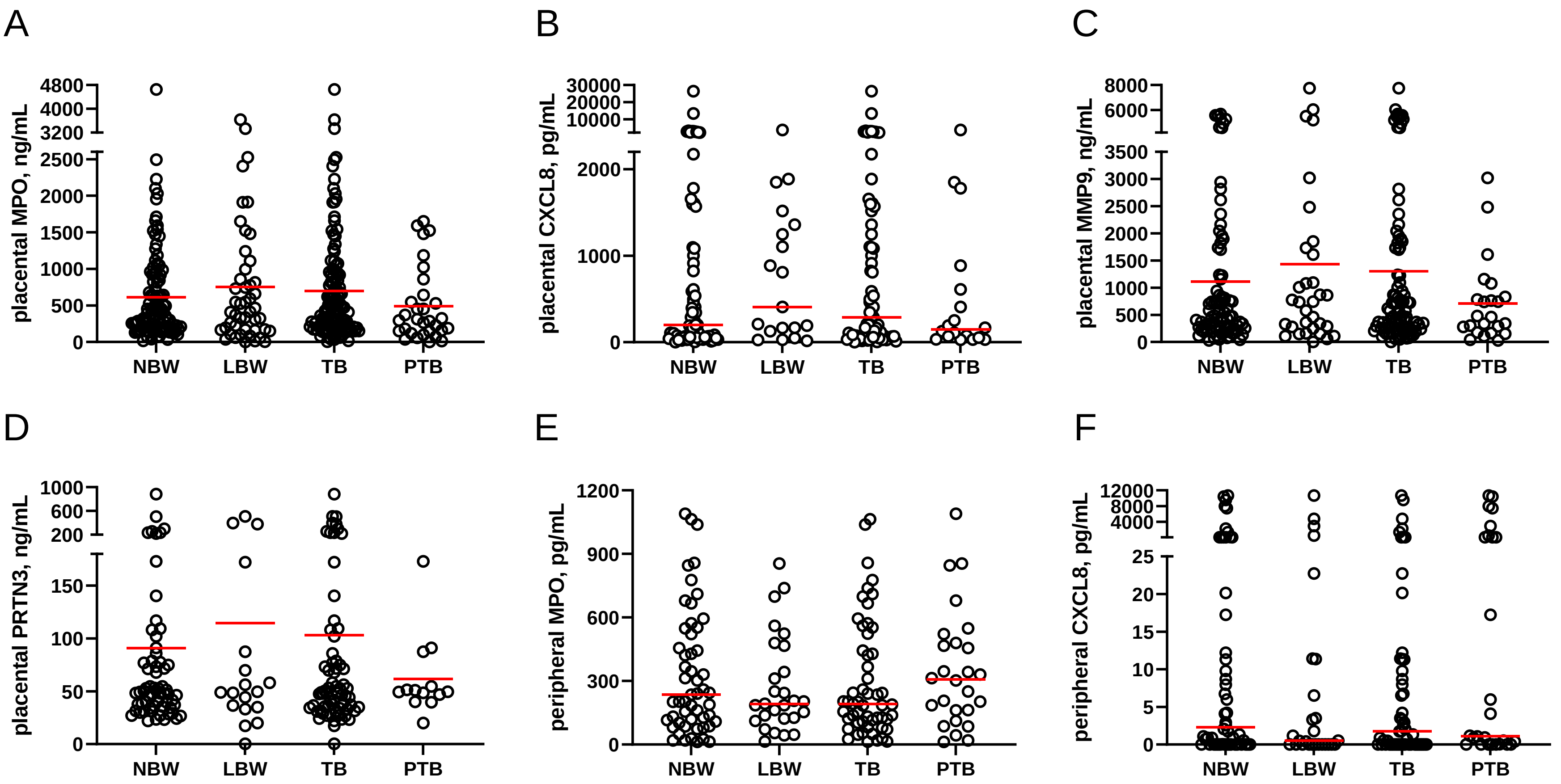

Supplemental figure 3. Infant outcomes are independent of peripheral and placental levels of markers associated with neutrophil activity.

Placental (A-D) and peripheral (E,F) plasma from paucigravid women was analyzed for A,E) myeloperoxidase (MPO), B,F) CXCL8, C) matrix metalloproteinase 9 (MMP9) and D) proteinase 3 (PRTN3) levels by bead array (or ELISA: CXCL8) and stratified by infant birthweight and gestational age at birth. No significant differences were evident by unpaired Mann Whitney U test among all analytes. NBW = normal birthweight (>2500 g); LBW = low birthweight (≤2500 g); TB = term birth (≥37 weeks); PTB (≤36 weeks).

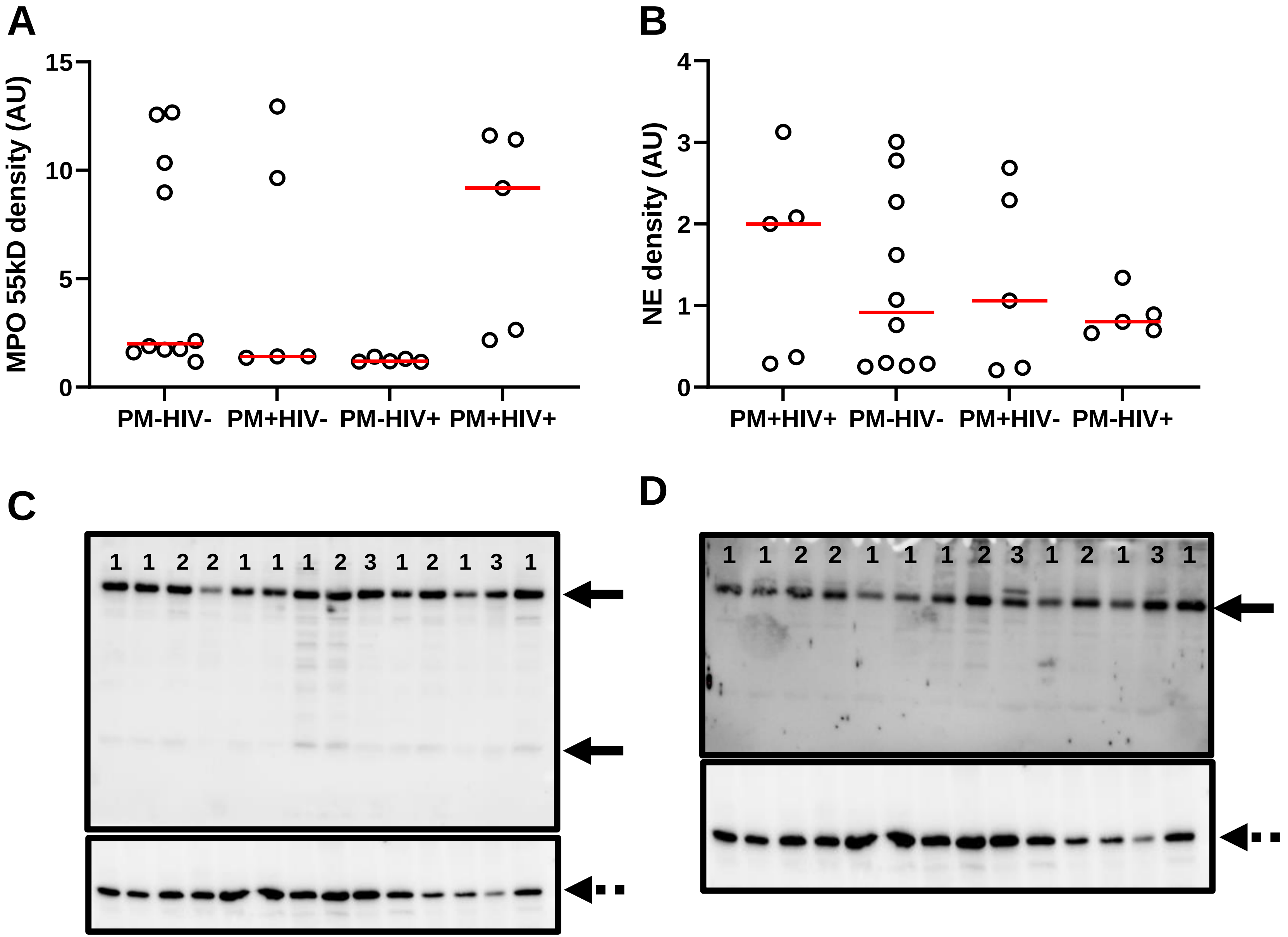

Supplemental figure 4. Detection of MPO by western blot.
A) MPO and B) neutrophil elastase protein levels do not differ as a function of infection status. Representative blots of C) MPO (55 KD and 15KD bands, non-dashed arrows) and D) HNE are shown. The dashed arrow indicates HSP90 protein as loading control. In C,D), 1, PM-HIV-; 2, PM+HIV-; 3, PM+HIV+. PM-HIV+ are in another set of data (not shown).

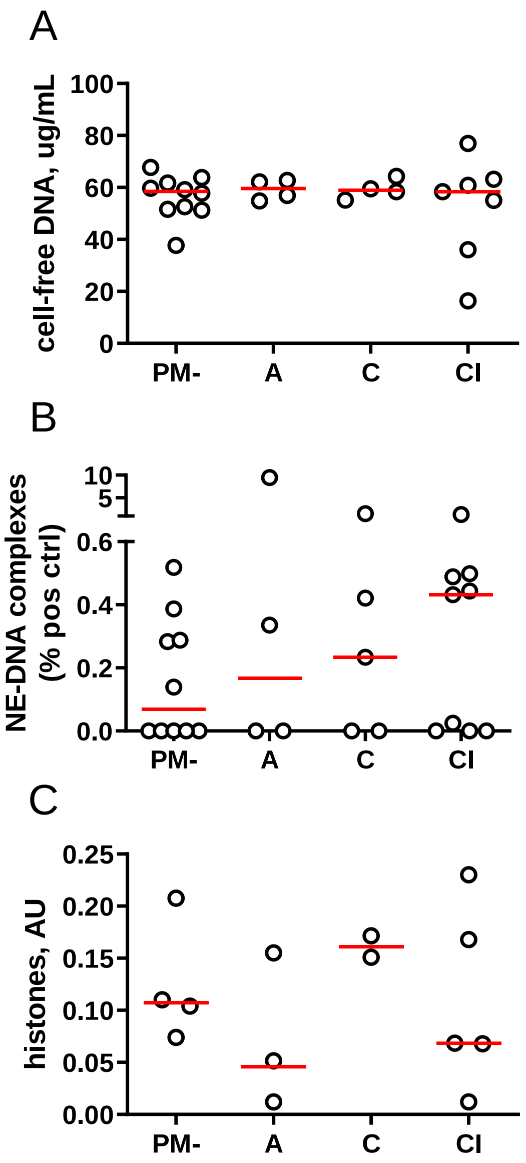

Supplemental figure 5. Plasma markers of NETosis do not differ as a function of placental histopathological status.
A) Cell-free DNA, B) neutrophil elastase-DNA complexes (HNE-DNA) and C) cell-free histones, all measured by ELISA in placental plasma from primigravid women. All *P* > 0.05 by Kruskal-Wallis test with post-hoc group-wise comparisons by Dunn's multiple comparisons test.

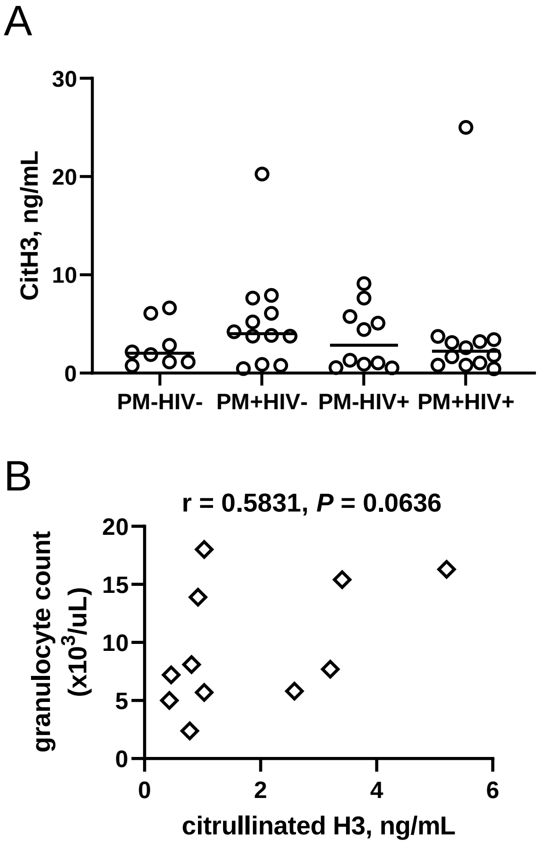

Supplemental figure 6. Placental plasma citrullinated histone levels weakly correlate with placental granulocyte counts but do not differ as a function of infection status.
A) Citrullinated histone 3 (CitH3) was measured by ELISA in placental plasma from paucigravid women with and without placental malaria and HIV infection. $P > 0.05$ by Kruskal-Wallis test with post-hoc group-wise comparisons by Dunn's multiple comparisons test. B) CitH3 levels tend to positively correlate with granulocyte counts in the placenta. Correlation by Spearman's test.

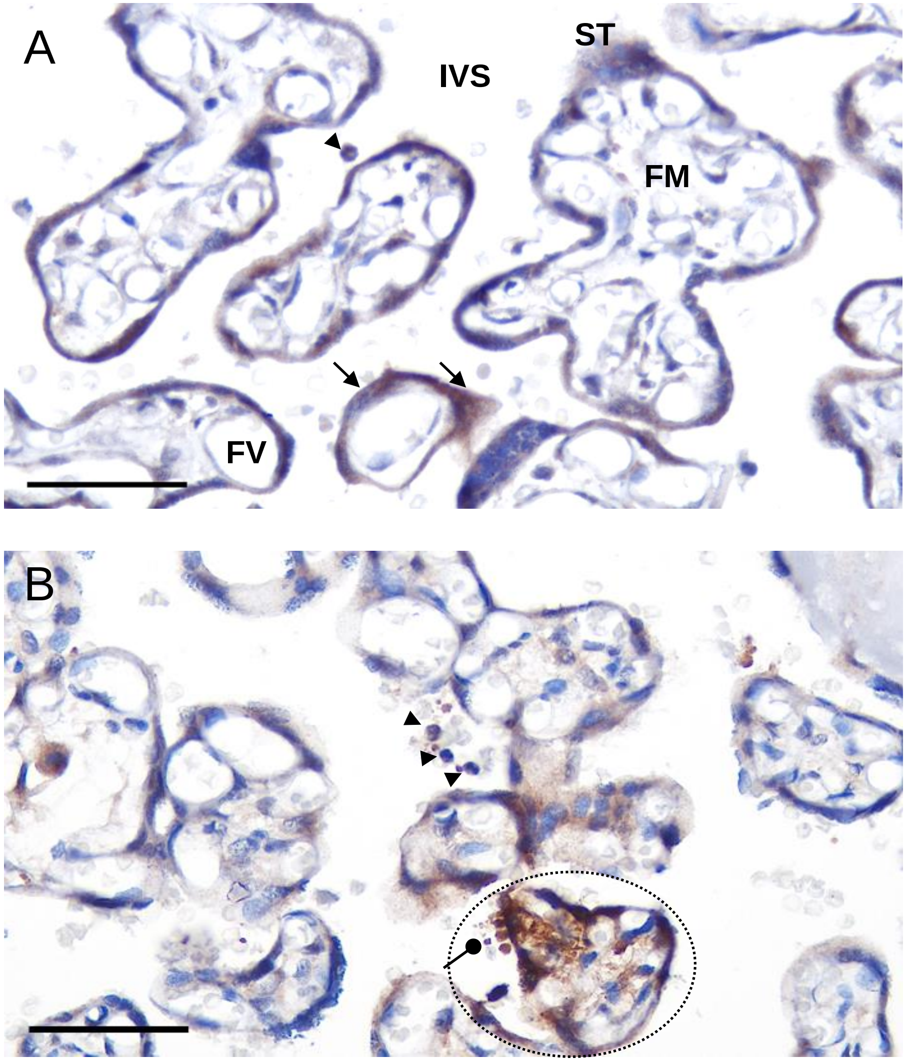

Supplemental figure 7. Citrullinated histone H3 (citH3) is mainly membrane bound.
A) Syncytiotrophoblast cytoplasm is moderately to strongly stained (in brown) for citH3 (black arrow). B) Syncytiotrophoblast is rarely focally strongly stained for citH3 cytoplasm with unapparent nuclei and cellular limits surrounded by pinpoint to round cellular debris (dotted circle) with a single degenerated neutrophil (round arrow). In both panels, neutrophils within the intervillous space are negative or lightly cytoplasmic stained for citH3 (arrowhead). ST: syncytiotrophoblast; FM: fetal mesenchyme; FV: fetal vessel; IVS: intervillous space. Scale bars represent 50 µm.

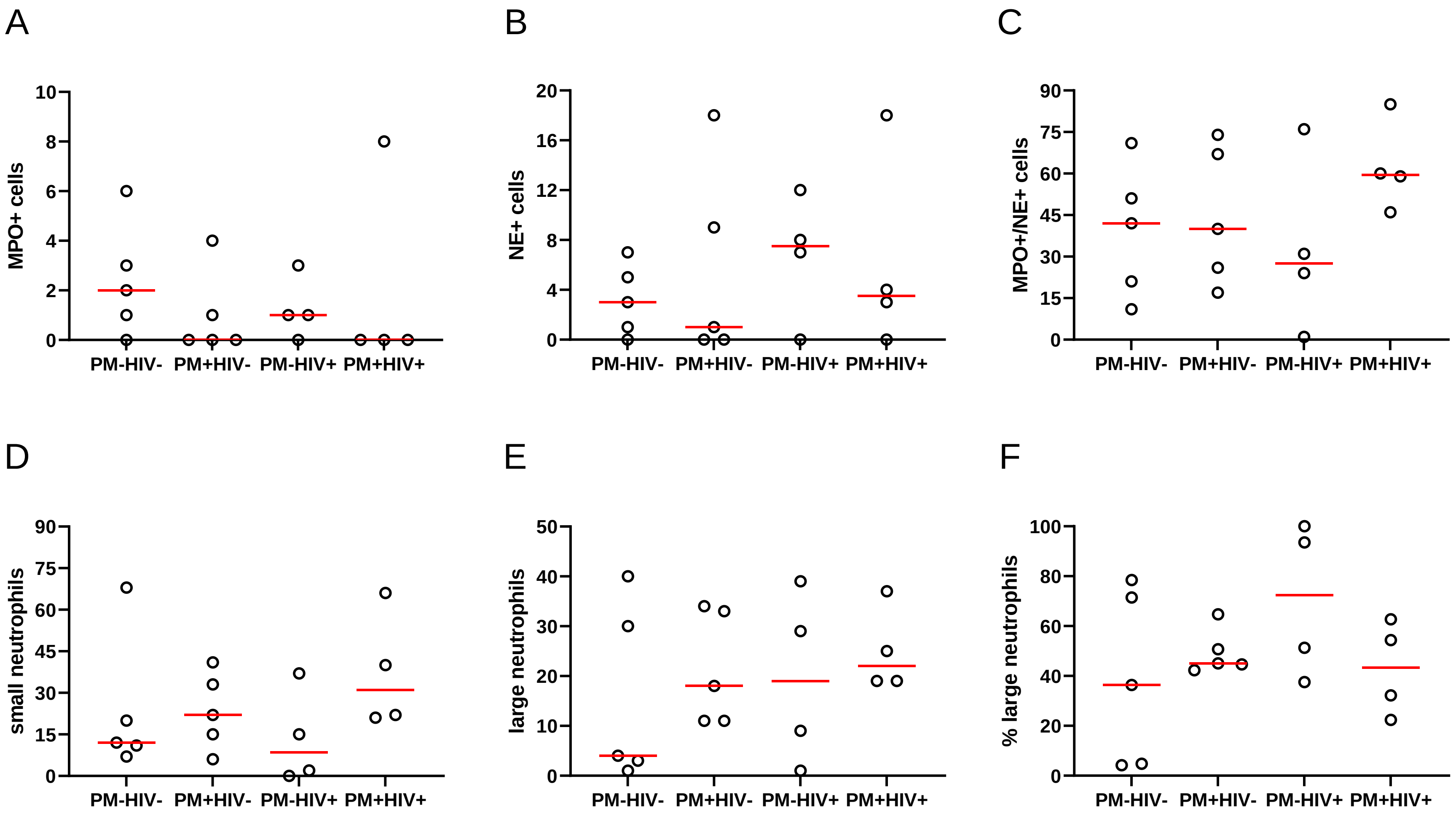

Supplemental figure 8. Detection of NETosis by immunofluorescence in frozen placental tissue. Counts of cells for A) MPO singly stained, B) neutrophil elastase (NE) singly stained, and C) MPO/NE doubly stained cells were enumerated by immunofluorescence across five fields of view in fresh-frozen placental tissue from paucigravid women with and without PM and HIV infection. Doubly stained cells were further stratified by size: D) small: <102 μm^2 , E) large: >102 μm^2, F) % of cells defined as large. The "large" definition is consistent with NETs. All $P > 0.05$ by Kruskal-Wallis test with post-hoc group-wise comparisons by Dunn's multiple comparisons test.

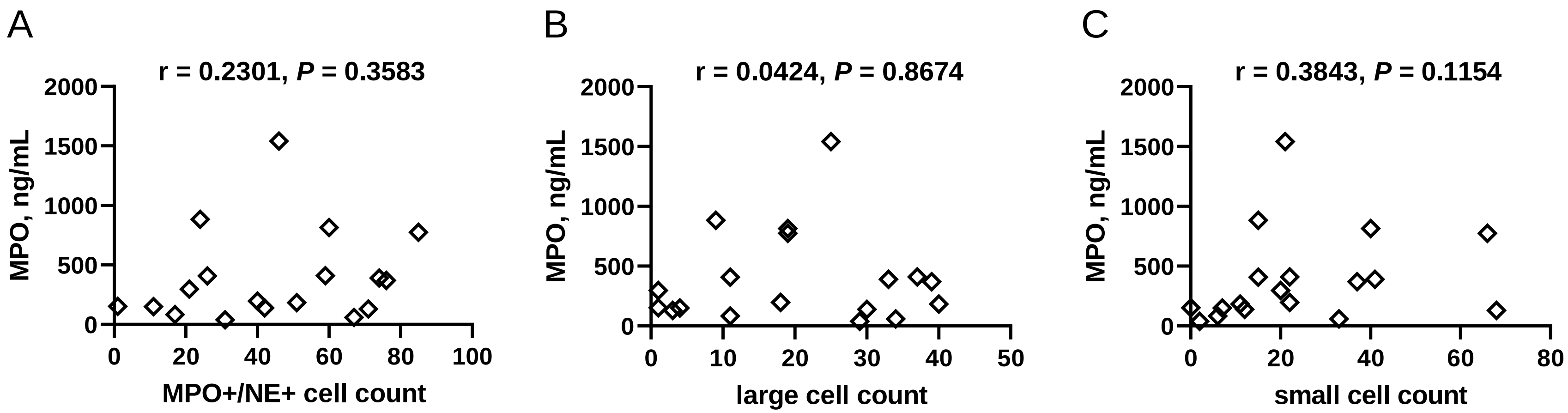

Supplemental figure 9. Correlation of neutrophils detected by immunofluorescence in frozen placental tissue with placental plasma MPO levels. Counts of cells for A) MPO/NE doubly stained cells were enumerated by immunofluorescence across five fields of view in fresh frozen placental tissue from paucigravid women with and without PM and HIV infection, considered collectively. Doubly stained cells were further stratified by size, B) large: >102 μm^2, and C) small: <102 μm^2, where the “large” definition is consistent with NETs. Correlation analysis by Spearman’s test.
